# Volcanism-induced collapse and recovery of the Atlantic meridional overturning circulation under glacial conditions

**DOI:** 10.1126/sciadv.adx2124

**Published:** 2026-02-04

**Authors:** Guido Vettoretti, Ruei-Jia Hu, Ingo Bethke, Kirstin Krüger, Michael Sigl, Stephen Outten, Jaimei Lin, Roman Nuterman, Anders Svensson, Peter Ditlevsen, Markus Jochum

**Affiliations:** ^1^ Physics of Ice, Climate and Earth, Niels Bohr Institute, University of Copenhagen, Copenhagen, Denmark.; ^2^ Department of Atmospheric Sciences, National Taiwan University, Taipei, Taiwan.; ^3^ Geophysical Institute, University of Bergen, Bjerknes Centre for Climate Research, Bergen, Norway.; ^4^ Department of Geosciences, University of Oslo, Oslo, Norway.; ^5^ Centre for Advanced Study, University of Oslo, Oslo, Norway.; ^6^ Oeschger Centre for Climate Change Research, University of Bern, Bern, Switzerland.; ^7^ Nansen Environmental and Remote Sensing Center and Bjerknes Centre for Climate Research, Bergen, Norway.

## Abstract

Volcanic eruptions have considerable impacts on climate across various timescales; however, it remains uncertain if, and how, volcanic activity could drive climate change over multiple millennia. Here we incorporate realistic volcanic forcing into a large ensemble of glacial era–coupled atmosphere-ocean model simulations. These simulations are constrained by sulfate records from ice cores, which help estimate the timing of past major eruptions. We investigate how volcanic eruptions may have occasionally triggered abrupt climate change during the last glacial period. Our results show that very large equatorial eruptions can induce large changes in the Atlantic Meridional Overturning Circulation via atmospheric and ocean circulation changes and air-sea buoyancy fluxes, potentially pushing the climate system between persistent warm and cold states lasting millennia. A simplified perspective of the dynamics shows how unforced natural climate variability may exert a stabilizing influence decades after an eruption, especially as the system nears a tipping point.

## Teaser

Introducing very large volcanic eruptions into a model of glacial climate causes abrupt climate change.

## INTRODUCTION

During the last glacial period from approximately 115,000 to 11,700 years ago (115 to 11.7 ka), Earth’s climate was marked by repeated, abrupt shifts between cold (stadial) and relatively warm (interstadial) phases, each lasting hundreds to thousands of years. The rapid transitions from cold to warm conditions are known as Dansgaard-Oeschger (D-O) warming events (*1*), and were most pronounced between 59 and 27 ka, corresponding to Marine Isotope Stage 3 (MIS 3). Ice-core analyses from Greenland reveal that these events involved dramatic temperature increases of 10° to 15°C occurring within just a decade or two (*2*). D-O events are considered key components of millennial-scale climate oscillations, and are thought to be linked to shifts between weak and strong states of the Atlantic Meridional Overturning Circulation (AMOC) (*3*). These often rapid transitions in ocean circulation are widely regarded as the primary driver of glacial millennial–scale climate variability, which are a prominent feature of the glacial-interglacial cycles of the Late Quaternary period. The underlying mechanisms driving these large-scale D-O oscillations are closely tied to changes in ocean circulation patterns and heat storage (*4*), which are often characterized as climate tipping points (*5*).

Past episodes of extreme and rapid climate change associated with D-O events represent major perturbations to the Earth system (*6*), and understanding their dynamics remains an active area of paleoclimatic research. They offer valuable historical context for potential climate instability driven by modern anthropogenic global warming and climate change. The 21st century is a critical juncture for humanity, prompting intense scientific interest in identifying climate tipping points and assessing the risks of irreversible impacts (*7*). In particular, the large AMOC shifts observed during past D-O events (*8*) provide a direct analog for one of the most concerning potential tipping points today, the collapse of the AMOC under anthropogenic climate change, which could have profound and long-lasting consequences for global climate this century (*9*). Understanding the role of volcanism within this framework is a critical component for future climate projections (*10*).

Cold stadials were characterized by extensive winter sea ice cover in the high-latitude North Atlantic, often extending as far south as the Bay of Biscay (*11*). During these cold stadial periods, the Atlantic Ocean continued to transport heat and salt into the high northern latitudes (*8*), but this heat was largely confined to subsurface waters beneath the expansive sea ice and strong halocline in the Labrador, Greenland, Irminger, and Norwegian Seas [collectively referred to as the Labrador Sea and Greenland, Norwegian and Irminger (GIN) seas] (*12*, *13*). Over centuries, this subsurface heat accumulated within the upper several hundred meters of the ocean, gradually increasing the heat content of the water column (*14*, *15*). Eventually, this buildup reached a critical threshold, triggering a sudden destabilization of the high latitude North Atlantic Ocean leading to an interstadial period (*12*–*14*). The abrupt release of stored oceanic heat to the atmosphere initiated a rapid warming phase (*4*, *15*, *16*), that reinitiated deep convection in previously ice-covered areas (*14*), effectively restarting the AMOC and reinforcing northward heat transport. This high Northern latitude warming created an amplifying feedback (e.g., sea-ice albedo feedback) that further accelerated high-latitude warming (*4*, *15*, *16*).

The increased atmospheric temperatures also intensified the hydrological cycle, leading to greater runoff into the Arctic (*16*). Eventually, this freshwater input, combined with enhanced sea ice export to the GIN seas, suppressed active convection and inhibited deep water formation (a key component of the thermohaline overturning circulation and AMOC) (*16*). Over the following centuries with a tapering off of the positive net radiation to space, the system gradually returned to a colder, more stable stadial state, marked by expanding sea ice cover in high latitudes. With the restart of the accumulation of subsurface ocean heat content in the North Atlantic (and negative net radiation to space), conditions would then be reset for another D-O event (*15*, *16*).

Complex coupled-climate modeling studies have shown that glacial millennial–scale climate variability can be influenced by a number of internal and external climate processes. Some of these include changes in incoming solar radiation variations associated with the Earth’s orbital dynamics (*15*, *17*), long term natural variations in atmospheric CO_2_ concentrations (*18*, *19*), glacial changes in continental ice sheet size (*20*), changes in runoff from land and ice sheets to the ocean surface that change the density structure of the ocean surface (*16*, *21*), as well as long-term changes in atmospheric variability (*15*, *22*–*26*). There have been several theoretical studies using simple models that demonstrate that D-O events are part of an internal climate oscillation influenced by forcing and/or noise processes (*27*–*30*). Theoretically, D-O events have been proposed as a feature of a simplified mathematical structure (a nonlinear dynamical system) that forms a basis for the complex climate system under glacial conditions (*19*, *31*–*33*). Comprehensive reviews of our understanding of D-O events are discussed in (*4*) and (*21*).

The potential role of volcanic eruptions in initiating or influencing millennial-scale climate variability has been discussed since at least the 1970s, although it has generally received limited attention (*34*, *35*). Mechanisms include the effect of volcanic ash and sulfate aerosol on planetary albedo, ocean fertilization, and phytoplankton productivity as well as causal volcanic activity induced through ice sheet unloading and crustal stress changes. Most studies proposing such a link rely on apparent temporal correlations between climate proxies and indicators of volcanic activity (*34*, *36*). However, these interpretations are constrained by three major uncertainties: (i) the accuracy of dating both climate and volcanic records, including estimates of sulfur emissions; (ii) the underrepresentation of past eruptions in available datasets; and (iii) the lack of process-based understanding connecting volcanic forcing to long-term climate responses.

Recent advances have helped address some of these limitations. Improved radiometric dating techniques have refined global volcanic records (*35*, *37*), while better interhemispherically synchronized polar ice-core data (*38*) and speleothem records (*39*) have enhanced the resolution of millennial-scale climate variations. Regardless, notable challenges remain. Few climate proxy records directly preserve evidence of tephra and sulfate deposition, making it difficult to attribute specific climatic responses to individual eruptions. Furthermore, regional case studies documenting post-eruption impacts cannot be confidently extrapolated across millennial timescales (*40*).

Studies based on rock and ice-core records (*41*) show that petrological estimates of stratospheric sulfur release often differ by orders of magnitude, yielding highly uncertain results. For example, estimates for the Youngest Toba Tuff eruption vary from 0 to 3000 TgS. Similarly, reconstructing accurate volcanic aerosol loads from ice cores is hindered by the lack of definitive links between sulfur anomalies and their source volcanoes as well as uncertainties in the atmospheric transport pathways that carried the aerosols to the ice-core sites. Further uncertainties arise from potential differences in aerosol scavenging efficiency during cold stadial versus warm interstadial periods. During stadials, generally drier phases, reduced precipitation limited the removal of aerosols from the atmosphere (*42*). In contrast, warmer interstadial conditions featured higher atmospheric water vapor content, which enhanced sulfate scavenging via increased precipitation along transport routes from eruption sources to ice-core locations.

Despite mechanistic uncertainties and the incomplete nature of many geological volcanic records affected by underreporting, spatial biases, and poor constraints on eruption magnitude (*41*, *43*, *44*), high-resolution ice-core records remain among the most reliable sources of pre-instrumental atmospheric aerosol history. A major advantage of ice cores lies in their dual preservation of both volcanic and climatic signals within the same archive, enabling direct comparisons between volcanic forcing and climate response. These archives not only constrain the timing and magnitude of stratospheric sulfate injections but also corecord key climate variables such as temperature, dust fluxes, and methane concentrations (*2*, *45*). Moreover, because the same ice cores used to derive volcanic sulfur emissions also contain high-latitude climate information with exceptional temporal precision, they allow analysis of the timing and state dependency of the climate response across a range of eruption parameters and background climatic states (*46*, *47*). Well-dated ice-core records offer continuous reconstructions of volcanic sulfate and ash layers over the past approximately 60 ka (*48*, *49*), with select records extending even further (*50*, *51*).

Large volcanic eruptions can inject substantial amounts of ash, dust, and sulfur dioxide (SO_2_) into the stratosphere, where they form sulfuric acid droplets (H_2_SO_4_ and H_2_O) that may persist for several years. These volcanic aerosols reflect incoming shortwave radiation, resulting in surface cooling of the Earth (*52*, *53*). The radiative impacts of volcanic activity on modern interannual climate variability have been well studied (*10*), including their influence on climate modes such as the El Niño–Southern Oscillation (ENSO) (*54*–*59*).

The influence of volcanism on ocean-atmosphere dynamics is known to extend beyond immediate cooling effects such as reductions in ocean heat content and increases in sea ice and glaciers (amplified by albedo feedbacks), for example, in relation to causation of the little ice age (*60*–*64*) and on modern climate variability (*65*, *66*). Multidecadal variability in the ocean’s climate can be influenced by cumulative volcanic activity from large eruptions as well as singular very large eruptions. Volcanic cooling increases Arctic sea ice extent, which further cools the high latitudes, enhancing sea ice growth (*63*, *67*). Climate simulations show that within the first 25 years of a high-latitude eruption, the AMOC strengthens because of surface cooling, reduced precipitation, and reduced runoff in the high North Atlantic region likely driven by ocean-sea ice feedbacks in high northern latitudes (*55*). The cooler more saline waters lead to increased densities that destabilize the water column and increase ocean deep convection. The initial cooling phase may also further intensify the AMOC over the subsequent decades due to increased sea ice formation and brine rejection in the high North Atlantic, which leads to saltier and denser waters (*68*).

Atmospheric teleconnections may also play an important role in modulating AMOC responses to volcanic activity. Recent studies that correlate AMOC variability and Irminger Sea density show strong control by the North Atlantic Oscillation (NAO) on multiple timescales (*69*, *70*). Furthermore, modeling studies suggest that cooling in the Tropical Pacific can strengthen the AMOC through Rossby wave propagation from equatorial to high northern latitudes, inducing sea level pressure adjustments in the subpolar gyre regions. Changes in circulation that result from the induced pressure changes increase the AMOC through changes in air-sea fluxes and ocean stratification as well as through increases in surface winds that modify the subpolar gyre strength (*71*). These responses are consistent with other studies linking AMOC variability to large-scale atmospheric teleconnections associated with sea surface temperature gradient changes in the tropical Atlantic (*66*, *72*) or changes in ENSO dynamics (*57*). Furthermore, volcanic activity affects energetic constraints on the position of the intertropical convergence zone (*73*) and would affect the balance between atmospheric and ocean energy transport through Bjerknes compensation. Climate simulations that introduce Holocene volcanic forcing demonstrate that 11 multicentennial cold periods in the Northern Hemisphere correlate with a clustering of enhanced volcanic activity (*74*). The clustering of volcanic events induces periods of multidecadal cooling (*63*, *68*, *69*, *75*) and may affect climate variability at the centennial and millennial timescale (*74*).

Understanding the full dynamical impacts of volcanic eruptions on the coupled atmosphere-ocean system during the glacial period requires more than paleoclimate proxy records alone. Because of the limited spatial coverage and imprecise chronological resolution of most paleoclimate archives, complex climate models are essential for simulating and interpreting the global-scale and long-term responses to volcanic forcing. Building on this foundation, the present study investigates whether volcanic eruptions could have acted as triggers or amplifiers of abrupt climate transitions during the last glacial period. By integrating high-resolution ice-core records with targeted climate model simulations, we assess the plausibility of a volcanic contribution to D-O–type variability, while also gaining insight into the resilience and internal dynamics of the glacial climate system.

## RESULTS

**Fig. 1.**
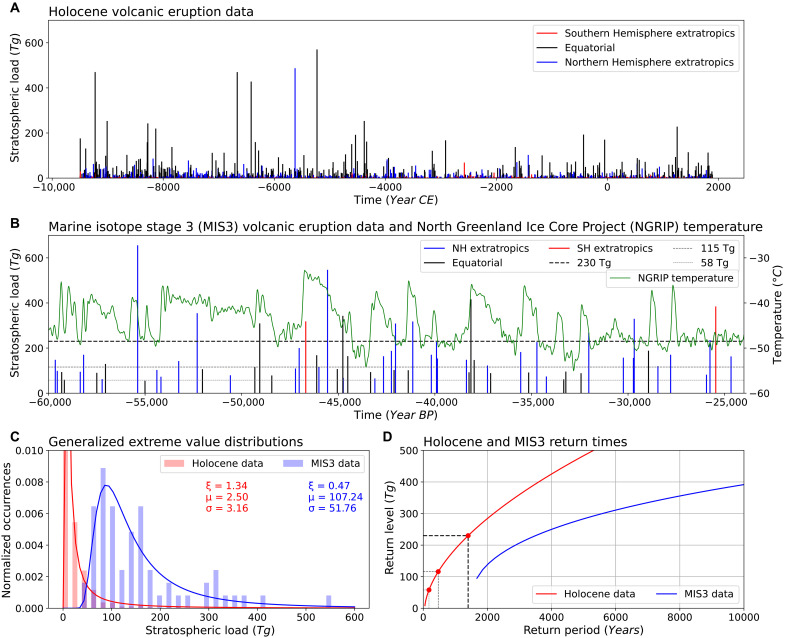
Records of explosive volcanism. Volcanism during (**A**) the Holocene (*49*) and (**B**) MIS 3 (*48*) with North Greenland Ice Core Project (NGRIP) temperatures in green (°C) (*111*). Horizontal dashed lines represent the volcanic forcing levels in the CCSM4 simulations from this study. (**C**) Time series in (A) and (B) are fit to a GEV. The GEV parameters are shape (ξ), location (μ), and scale (σ). (**D**) The return level is the volcanic eruption size over the specified period of time before reoccurrence, the return period. See Supplementary Text.

Time series of estimated stratospheric loading in teragrams (Tg) of sulfate (SO_4_) for both the Holocene (11.7 ka to present) (*49*) and MIS 3 (*48*) based on ice core records are shown in Fig. 1 (A and B), respectively. During MIS 3, most D-O events with a well-dated chronology appear frequently in ice-core climate records from Greenland and Antarctica. Here, in this study, we model the frequency distribution of large to very large volcanic eruptions [such as 1991 Pinatubo, 1815 Tambora, and 1257 Samalas; (*76*–*78*)] during the Holocence and MIS 3 periods using a generalized extreme value (GEV) distribution.

The GEV distribution is a family of continuous probability distributions developed within extreme value theory. It is commonly used to model the maximum (or minimum) values observed over fixed time intervals, for example, annual maximum daily rainfall or coldest temperature each year. GEV probability distributions have “fat tails” that capture the probability of rare but impactful events. In our analysis the frequency of volcanic events align well with a GEV probability distribution, except for an underestimation at higher values, corresponding to longer intervals between events (see Fig. 1C).

The calculated return times for a 230-Tg volcanic aerosol event (Fig. 1D), such as is used in this modeling study, are generally in the range of 1500 years for the Holocene and more than 3000 years for the MIS 3 period, with the Holocene analysis showing more frequent occurrences of large events. The actual amount of volcanic sulfate (SO_4_) in the climate model aerosol mass load would equate to a smaller amount of sulfate deposition (≈217-Tg SO_4_) observed in the ice cores due to the presence of water in the modeled aerosols (*79*). Therefore, the 230-Tg return levels (Fig. 1D) as recorded in the ice cores corresponds to a slightly more frequent return period, closer to 1000 years.

The differences in return periods for a given return level between eras stem from the methodological differences in reconstructing SO_4_ load from ice cores. These differences arise from choices in data reconstruction methods, such as smoothing records to the lowest resolution (for MIS 3) or combining multiple records (for the Holocene). Because of the challenges in obtaining a consistent volcanic forcing record from the glacial period, the return periods for MIS 3 may be overly conservative. The thinning of the annual layers in ice cores with depth affects the signal-to-noise ratio deeper into the glacial period. In contrast, return periods for the Holocene are considered to more accurately reflect the true probability of eruptions in the 100- to 500-Tg SO_4_ range. The return time analysis provides a framework for assessing the impact of very large volcanic eruptions on glacial period climate within a complex climate model.

The model used in this study is the Community Climate System Model version 4 (CCSM4), which is part of the National Center for Atmospheric Research Community Earth System Model initiative (see Materials and Methods). We use a lower-resolution version of this coupled atmosphere-ocean model (*80*) to run long multi-millennial glacial climate simulations that would otherwise be computationally prohibitive using a model with resolution consistent with those used in the Climate Model Intercomparison Project (CMIP), which are run at higher resolution. In the CCSM4 model, the maximum global cooling immediately following a very large equatorial Samalas-like (*77*, *78*) volcanic eruption (230 Tg volcanic aerosol) remains relatively constant irrespective of modern or glacial climate (stadial or interstadial) background conditions. The global average cooling anomaly reaches a peak value of approximately −2.5°C (fig. S5) and is consistent with other climate models (*81*–*83*). This equates to a mean global maximum aerosol optical depth (AOD) of approximately 1.75 at 550 nm a few months following the volcanic eruption (fig. S1). In our simulations, volcanic events start on January 1st and peak in July of the same year, and, therefore, the AOD forcing is affected by a seasonal bias in incoming solar radiation (fig. S2). However, the radiative forcing associated with these simulations are equatorially confined and not dynamically advected, so seasonality is not expected to influence the results with this model.

The chronology of MIS 3 has previously been constrained using bipolar volcanic matching to identify tie points in ice-core records from Antarctica and Greenland (*38*). Numerous sulfate deposition anomalies are preserved in these ice cores, many of which correspond to extratropical volcanic eruptions that are key to understanding past climate variability. Examples include the 7.6-ka Crater Lake (Mazama) eruption in Oregon (*74*, *84*) and the 25.3-ka Oruanui supereruption from New Zealand’s Taupō Volcano (*85*, *86*). It is well documented that large caldera–forming eruptions in the Northern Hemisphere mid-latitudes (e.g., Okmok, Aniakchak, and Crater Lake) can result in detectable sulfur deposition in both polar regions, with Antarctic ice cores recording some of the largest sulfate signals of the Holocene (*49*). Studies have shown that eruptions of high magnitude and plume height, despite their extratropical origin, can exhibit interhemispheric transport characteristics similar to those of tropical eruptions, although the spatial distribution remains more asymmetric (*87*–*89*).

While the volcanic source attribution used here accounts for such behavior (*48*), future investigations into the climate responses should incorporate more detailed analyses of seasonal and hemispheric variations in volcanic forcing. Moreover, in simulations of D-O events with hemispherically confined volcanic forcing, one might also anticipate modulations due to the bipolar seesaw effect (*90*), wherein cooling in one hemisphere may be accompanied by warming in the other, a response that is well documented and inherent to glacial climate systems. Analyzing the climatic impact of such large extratropical eruptions is beyond the scope of this study as the aerosol forcing in our model is prescribed rather than dynamically advected. This aspect is therefore reserved for future research.

**Fig. 2.**
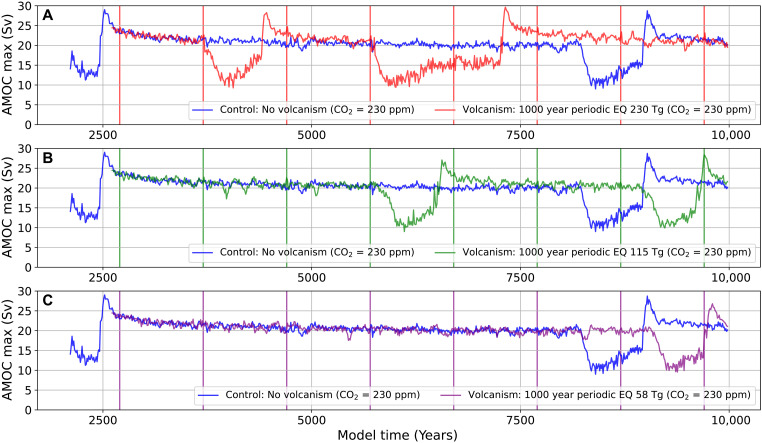
The D-O cycle under volcanic forcing. The response of the D-O cycle to periodic equatorial volcanic forcing under glacial climate conditions, with the control simulation in blue overlaid with the experiments in red, green and purple. Each experiment is forced every 1000 years with volcanic events and branched off the control at year 2601. Volcanic events are marked by the vertical lines releasing (**A**) red: 230-Tg volcanic aerosol (AOD max = 1.74), (**B**) green: 115-Tg volcanic aerosol (AOD max = 0.91), and (**C**) purple: 58-Tg volcanic aerosol (AOD max = 0.49).

To understand the glacial climate response to equatorial volcanic forcing, we use an initial sensitivity study with periodic volcanic forcing of 230-Tg volcanic aerosol every 1000 years to examine the impact of volcanism on the D-O cycle. We also investigate how a reduction in the magnitude of the equatorial volcanic forcing from 230- to 115- and 58-Tg volcanic aerosol affects the results (Fig. 2). As we reduce the magnitude of the volcanic forcing, the response of the D-O cycle to volcanic forcing appears very muted below 115-Tg volcanic aerosol (Tambora-like eruption). When the volcanic forcing is at 58-Tg volcanic aerosol (Pinatubo-like eruption), the climate system response is indiscernible from random fluctuations associated with internal natural variability.

The behavior observed in the periodic volcanic forcing simulations appears to be probabilistic in nature. During the slow decay of the D-O interstadial over millennial timescales, evolving climate conditions associated with internal climate variability can give rise to decadal-scale states in which the AMOC is unusually strong. In such cases, the system may exhibit little or no response to an applied volcanic forcing. This suggests that the climate system’s response to volcanic perturbations is not deterministic but rather depends on the background state and internal variability (i.e., “noise”) at the time of the eruption. For instance, tipping behavior may occur during one phase of the interstadial cycle yet be absent under seemingly similar AMOC conditions at other times within the same D-O cycle. This is evident when comparing volcanic events occurring at years 3701 and 8701 in the equatorial 230-Tg volcanic aerosol experiment (Fig. 2A). Furthermore, the initial phase of the interstadial, characterized by an AMOC overshoot during the transition from cold stadial to warm interstadial (a D-O warming event), renders the system more resilient to volcanic eruptions that might otherwise trigger a shift back to a cold state. These findings underscore the importance of conducting very large ensembles of climate simulations to draw robust statistical conclusions about the behavior of the climate model under volcanic forcing.

### Volcanic impacts on interstadial and stadial climate

**Fig. 3.**
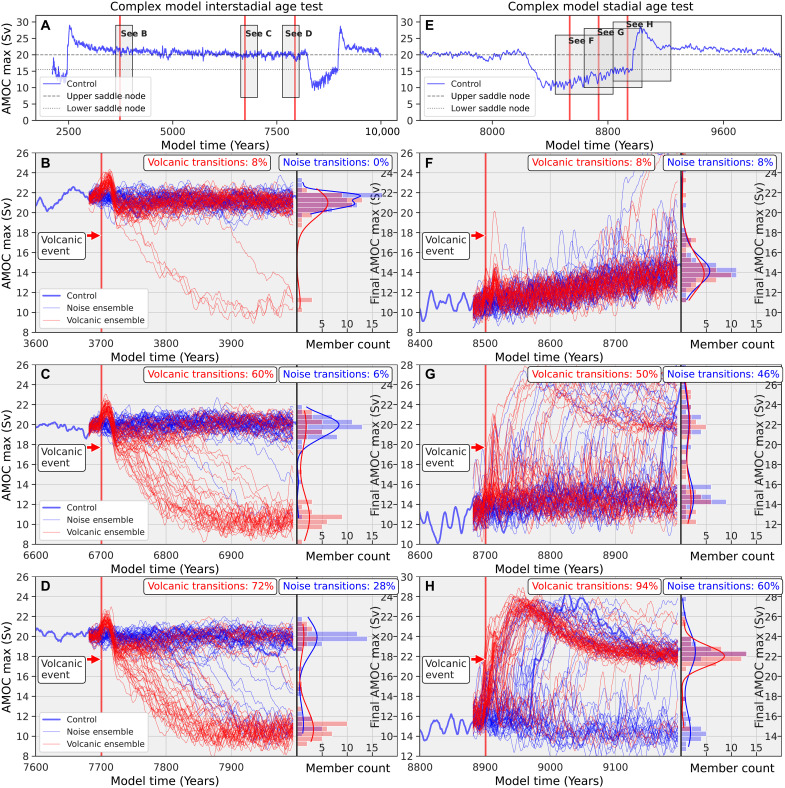
CCSM4 interstadial and stadial transitions probabilities. For AMOC collapse (left) and AMOC recovery (right) due to an equatorial volcanic eruption. (**A** and **E**) Control simulation. (**B** to **D**) Interstadial analysis: volcanic forcing ensemble (red) and no volcanic forcing ensemble (blue) for years 3701, 6701, and 7701. (**F**) to (**H**) are the same as in (B) to (D) but for the stadial analysis at years 8501, 8701, and 8901. The histograms and kernel density estimation represent the distribution of the AMOC maximum 300 years after the volcanic perturbation.

A D-O simulation with typical values of CO_2_ observed during the early MIS 3 period [55 ka, CO_2_ = 230 parts per million (ppm)] is used as a benchmark (*19*) to spin off ensemble members to investigate the probabilistic impacts of large single volcanic events on the glacial climate system (the control simulation in Fig. 2). This control simulation AMOC is characterized by a 5000-year interstadial period (Fig. 3A) followed by a much shorter stadial period (Fig. 3E).

For the following analyses, we use a 50-member CCSM4 ensemble without volcanic eruptions and a 50-member CCSM4 experiment ensemble that includes very large equatorial volcanic eruptions at six different periods of a D-O oscillation for a total of 600 simulations (gray boxes in Fig. 3, A and E). Each member in an ensemble has the initial surface temperature field from the long control simulation perturbed by a random infinitesimal value to understand the impact of both natural internal climate variability (the runs without the volcanic forcing) and the one-time large climate perturbation experiments (the runs with the volcanic forcing at different points of the D-O cycle (see Materials and Methods).

The modeled climate system is hypothesized to have two quasi-stable states: one characterized by a strong AMOC and the other by a weaker AMOC, a concept that has garnered a great deal of recent attention in relation to anthropogenic climate change (*5*). Dynamical systems that model the AMOC can undergo spontaneous transitions between these states solely due to internal noise or stochastic perturbations (*91*). To assess the stability of the AMOC under volcanic forcing in this model, we analyze a large ensemble of simulations forced with 230-Tg of volcanic aerosols (red simulations), as well as an equivalent ensemble without volcanic forcing (blue simulations), initiated at various phases of both the interstadial and stadial periods (vertical red bars in Fig. 3, A and E).

As the long multi-millennial control simulation (blue curve) approaches a tipping point, it is expected to become increasingly susceptible to (climate) noise-induced transitions, assuming that the variance of internal climate variability remains relatively constant over time. This behavior provides a test of our hypothesis that the glacial climate model reflects an underlying two-state AMOC dynamical system structure, as previously proposed (*19*). While this study aims to attribute a fraction of the abrupt climate changes of the glacial period to large volcanic perturbations, it also offers insights into long-standing questions regarding the fundamental structure and dynamics of the glacial climate system.

Each simulation in both ensembles includes a random perturbation of magnitude 10^−14^ K added to the surface temperature field 25 years before the onset of the volcanic event. The blue simulations, also initialized with small perturbations, allow us to estimate the probability of noise-induced transitions, while the red simulations provide estimates of the volcanic-induced transition probability. During the interstadial phase, the behavior of the ensemble pairs indicates the presence of multiple equilibria. This is evident in the increasing likelihood of noise-induced transitions over time, as shown in the blue simulations. The volcanic simulations similarly suggest bistability, indicating that the barrier separating the two AMOC states diminishes as the system nears a tipping point. Since volcanic perturbations are much stronger than background climate noise, they lead to higher transition probabilities.

The stadial ensemble simulations also exhibit similar behavior to the interstadial cases but are characterized by a pronounced AMOC overshoot as the climate transitions from cold to warm conditions. During the midstadial phase, noise-induced transitions show a similar propensity to volcanically forced transitions in shifting the climate system to the upper (strong AMOC) state (Fig. 3G). Intuitively, this reflects a greater degree of climate system stability in the warm period, which is evident from the system’s tendency to reside in the warmer state for millennia, considerably longer than in the colder state (Fig. 2). Another notable feature is that during the late interstadial, when the system is most susceptible to tipping, many simulations in the volcanic ensemble either fail to transition or fully recover after an initial shift toward the cold state on the multidecadal timescale. This behavior suggests that unforced natural climate variability can modulate the likelihood of a transition occurring under volcanic forcing.

Another prominent feature of the climate response to volcanic forcing is the initial strengthening of the AMOC immediately following the eruption, followed by a decline below the mean unforced AMOC level in both the interstadial and stadial periods, occurring within two decades. This transient behavior may play a critical role in driving the climate system across its tipping point. The physical mechanisms underlying this initial response are discussed below, while the longer-term, multidecadal evolution of the climate system is examined later through the framework of dynamical systems theory.

### Postvolcanic teleconnections and AMOC strengthening

The volcanic perturbations used in this study are substantially larger than any documented eruptions during the modern observational period. The impact of such high-magnitude volcanic forcing has primarily been studied in the context of the modern climate system. However, the presence of massive continental ice sheets in the complex model strongly influence albedo and atmospheric dynamics and necessitates an in-depth analysis of the atmosphere-ocean response to these extreme volcanic events. We should not expect the same teleconnections or responses that have been documented in studies of the modern climate in response to volcanic forcing, although we make note of connections with previous studies if they prove relevant.

An analysis of the immediate postvolcanic (year 7701) strengthening of the AMOC is a critical aspect of the system’s response and subsequent relaxation, either into a weakened state or toward a stable recovery. Following the initial strengthening, the AMOC may either weaken and transition to a weaker equilibrium or decay back to its preeruption state. Understanding this initial strengthening is essential as it represents a central component of the multidecadal postvolcanic stability of the AMOC.

Following a very large volcanic eruption, the planet experiences a radiation deficit that coincides with pronounced global cooling during the first few years after the event (fig. S6). This cooling is a direct consequence of increased AOD in the atmosphere, primarily due to sulfate aerosols injected into the lower stratosphere. The global cooling is also associated with changes in the hydrological cycle, including a drier atmosphere and enhanced sea ice growth at high latitudes in the North Atlantic, as discussed below.

To understand the sequence of events linking a volcanic eruption to changes in the AMOC and meridional heat transport in the North Atlantic, we first provide context for the mechanisms described in the following sections. The simulations reveal that the AMOC initially strengthens and then weakens over a period of a couple of decades, a pattern that raises the immediate question of which Earth system mechanisms play critical roles in this response. All ensemble members that include a volcanic event show a consistent initial strengthening of the AMOC. However, in the subsequent decades, the trajectory diverges; some ensemble members transition to a cold climate state, while others remain in a warm state, with this divergence appearing largely stochastic.

Therefore, our analysis adopts two complementary approaches. To understand the initial AMOC strengthening, we examine the ensemble-mean behavior. To understand the longer-term evolution, specifically, why some ensemble members transition to a cold state while others do not, we used a probabilistic framework based on a simple dynamical systems model. This approach helps isolate the lower-order dynamics of the climate system and provides insight into the underlying causes of the divergent responses.

In the first exercise, a central question arises immediately: How can a change in radiative forcing caused by a volcanic eruption translate into a change in ocean circulation? As noted in the introduction, previous studies have already offered insight into some of the mechanisms that may underlie this connection. Here, we build on that foundation by presenting our general interpretation based on model results of the sequence of events leading to the initial increase in AMOC strength.

This initial AMOC strengthening coincides with buoyancy loss in the high-latitude North Atlantic during winter, driven by positive sea surface salinity (SSS) anomalies in the same region. In the years that follow, however, the AMOC weakens. This subsequent decline is linked to increased buoyancy input at the surface of the North Atlantic Deep Water formation regions, occurring in both summer and winter. This buoyancy gain is attributed to enhanced sea ice growth, the development of a pronounced halocline, and changes in wind stress over the North Atlantic subpolar gyre, which strengthens immediately after the eruption. The intensified gyre circulation could enhance thermohaline circulation through increased meridional salt advection from the equator toward the pole.

The emergence of a negative NAO pattern is also discussed. However, we do not propose a direct cause-and-effect relationship here; rather, the global cooling may simply trigger a reorganization of internal modes of climate variability (e.g. such as ENSO, the Pacific Decadal Oscillation, and the NAO) that are complementary to the observed changes. For instance, the initial increase in SSS can be expected under a negative NAO phase, which reduces the meridional density gradient and thereby weakens moisture transport into the Norwegian Sea, a key region for NADW production.

In a first step to illustrate the AMOC response in terms of climate forcing and feedbacks, we analyze the thermal and haline contributions to ocean surface fluxes over the first two decades after the eruption. The total ocean surface heat flux (e.g., shortwave and longwave radiation, latent and sensible heat fluxes, etc.) determines the vertical thermal buoyancy flux at the surface of the high-latitude North Atlantic Ocean. The total surface freshwater flux (precipitation, evaporation, runoff, sea ice brine rejection, etc.) determines the vertical haline buoyancy flux. Wind stress also plays a key role in postvolcanic circulation changes and is described below. Wind stress primarily influences the wind-driven gyre circulation, whereas thermal and haline buoyancy fluxes drive the thermohaline component of the AMOC, which is crucial for its stability.

**Fig. 4.**
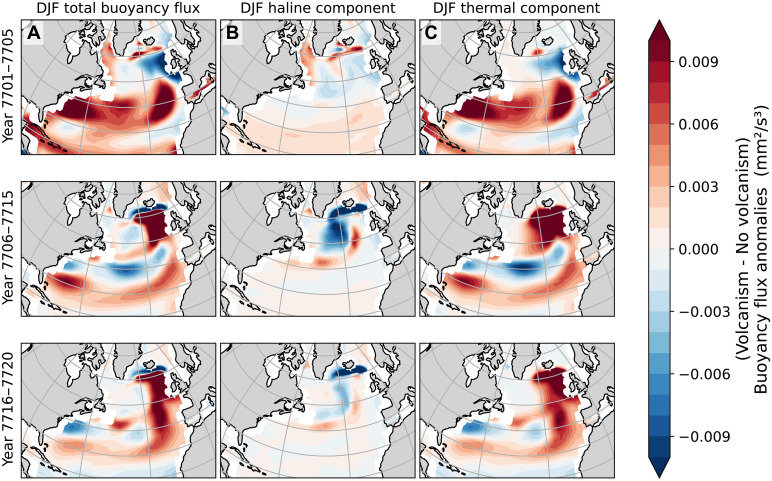
Winter surface buoyancy flux anomalies. The (**A**) total, (**B**) haline, and (**C**) thermal surface buoyancy fluxes anomalies. Time runs forward from the top to the bottom panels. Positive anomalies indicate that the ensemble average with the volcanic event results in a more buoyant ocean surface and more resistant to deep water formation associated with the AMOC.

**Fig. 5.**
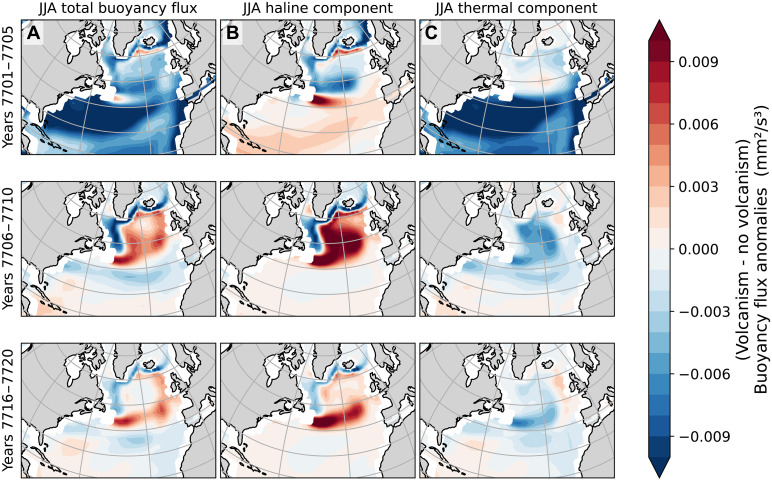
Summer surface buoyancy flux anomalies. The (**A**) total, (**B**) haline and (**C**) thermal surface buoyancy fluxes anomalies. Time runs forward from the upper to the lower panels. Positive anomalies indicate that the ensemble average with the volcanic event results in a more buoyant ocean surface, and more resistant to deep water formation associated with the AMOC.

The winter ensemble average total surface buoyancy flux anomaly, evaluated across three time periods following the volcanic eruption at year 7701 (Fig. 4A), aligns temporally with the initial strengthening followed by a weakening of the AMOC. Anomalies in Fig. 4 (winter) and Fig. 5 (summer) represent the ensemble average with volcanism minus the ensemble average without volcanism. Positive (negative) anomalies indicate buoyancy input (removal) into the ocean surface.

In winter immediately after the eruption, the high-latitude North Atlantic, particularly the Norwegian Sea, experiences a removal of buoyancy (which would enhance deep water formation and AMOC strength). In summer, negative buoyancy flux anomalies extend more broadly across northern latitudes. Over time, positive buoyancy flux anomalies emerge over the subpolar gyre region of the North Atlantic during summer. The initial summer buoyancy loss appears to be driven by the haline component rather than the thermal one. In contrast, the early winter response is dominated by the thermal component of the buoyancy flux.

**Fig. 6.**
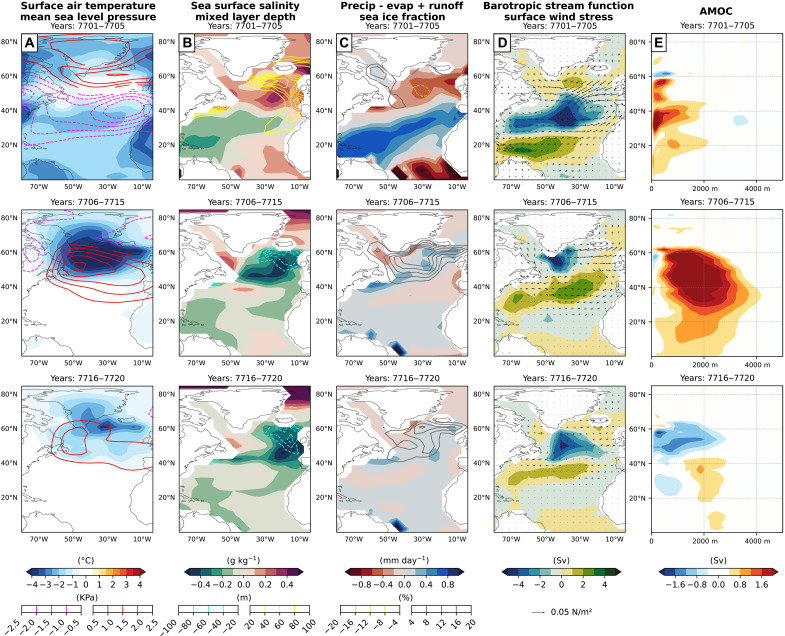
Postvolcanic North Atlantic climate changes. Time sequence (rows) of North Atlantic ensemble mean climatological anomalies (volcanism - no volcanism). Filled contours from left to right: (**A**) 2-m surface air temperature, (**B**) SSS, (**C**) P − E + R, (**D**) barotropic streamfunction, and (**E**) the AMOC anomaly. Line contours from left to right: (A) mean sea level pressure, (B) mixed layer depth, and (C) sea ice fraction. (E) The AMOC anomaly is matched in latitude with the other metrics in the columns. (D) Surface wind-stress is depicted as a vector plot. Negative line contours are dashed. Time sequences flow from top panels to bottom panels spanning years 7701 to 7705, 7706 to 7715, and 7716 to 7720 after the volcanic eruption.

However, deep water formation associated with the AMOC predominantly occurs during winter, suggesting that the initial strengthening of the AMOC is linked to the enhanced winter ocean-to-atmosphere heat fluxes (a loss of buoyancy). A decade after the eruption (years 7705 to 7715), increased buoyancy flux into the ocean surface in deep-water formation regions appears to stem from higher thermal buoyancy flux in winter and increased haline buoyancy flux in summer (see time slices in Figs. 4C and 5B). To better understand how these seasonal buoyancy fluxes are influenced by individual climate components, we examine the respective physical processes that contribute to thermal and haline components (Fig. 6).

Several notable differences emerge between the ensemble mean of volcanic simulations and those without volcanic forcing. The annual-mean changes in the North Atlantic result largely from widespread global atmospheric cooling following the volcanic event. An El Niño pattern develops during the first two winters following the volcanic event, although it is superimposed on a strong background cooling in the tropical Pacific (Supplementary Text, figs. S6 and S9). This suggests potential contributions from both tropical–high latitude teleconnections that reorganize North Atlantic pressure systems and the direct response of the high-latitude North Atlantic to the global cooling that follows the eruption (Fig. 6A). A Pacific–North American pattern (fig. S7) emerges in the Northern Hemisphere (*71*), characterized by distinct pressure anomalies over the North Pacific and North Atlantic. Specifically, the Icelandic Low and the Azores High exhibit an anomalous pressure increase and decreases, respectively, corresponding to the development of a negative NAO (Supplementary Text, figs. S9 and S11) in the first few years after the eruption. Therefore, the Icelandic Low and the Azores High both weaken. This reduces the meridional pressure gradient and reduces the westerlies, which bring moisture into the high-latitude North Atlantic following the volcanic eruption.

In the immediate aftermath of the eruption (years 7701 to 7705), this negative NAO phase is accompanied by anomalously reduced precipitation and runoff over the GIN Seas, which lowers freshwater input to the ocean and thereby increases SSS in key deep-water formation regions of the Irminger and Norwegian Seas (Fig. 6B). The volcanic-induced rise in SSS stems from a reduction in net freshwater flux (P – E + R, i.e., precipitation minus evaporation plus runoff) over the high-latitude North Atlantic (Fig. 6C), particularly within these deep-water formation zones. Surface cooling over these SSS anomalies would coincide with a drier high-latitude atmosphere, further suppressing P – E + R; however, this seems to be a secondary effect as the negative temperature anomalies are not large over this region (Fig. 6A). Studies linking volcanic aerosols to the AMOC changes in the modern climate have noted similar effects at high latitudes (*55*, *92*).

Some studies have shown that the wintertime NAO tends to strengthen in the aftermath of volcanic eruptions in the modern climate system (*69*). However, the simulations presented here are conducted in a glacial climate regime featuring extensive North American ice sheets that modify the structure of pressure patterns over the North Atlantic compared to the modern climate (Supplementary Text, figs. S8, S9 and S10), and we cannot expect the same response seen in the simulations using modern climate conditions.

Other notable change include concurrent shifts in sea level pressure and wind stress (Fig. 6D) that strengthen the North Atlantic subpolar gyre during this onset of initial AMOC strengthening (*22*). This intensified circulation enhances the northward transport of saltier subtropical waters via the Gulf Stream, delivering additional salinity to convection sites near Greenland and reinforcing the AMOC through a positive feedback mechanism (fig. S13).

Subsequently, anomalous decreases in SSS and slight increases in P − E + R over the deepwater formation regions of the GIN seas are seen in the decade following the eruption in years 7706 to 7720 (Fig. 6, B and C, respectively). The SSS decrease correspond with increased thermal buoyancy input into the GIN seas in winter and increased haline buoyancy input in summer (see Figs. 4C and 5B, respectively). This may be related to ocean surface warming anomalies in winter and enhanced winter sea ice growth that partially melts in the summer period. During this multidecadal period, sea ice expands over the primary deep-water formation regions due to intense high-latitude cooling (Fig. 6C). This leads to a more stable stratification, marked by the formation of a large-scale halocline that suppresses convection and initiates a feedback loop wherein the AMOC weakens further (*62*). As a result, the AMOC drops below the levels characteristic of the nonvolcanic control ensemble within about a decade after the eruption (Fig. 3, B to D) (*69*).

In summary, the volcanic eruption triggers a cascade of atmosphere-ocean interactions in the North Atlantic. Initially, during the first few years (years 7701 to 7705), global cooling modifies tropical-extratropical teleconnections and induces a negative NAO phase. This weakens the meridional pressure gradient, reduces moisture transport into high latitudes, and lowers freshwater input (P – E + R) to the GIN seas, thereby increasing SSS in deep-water formation regions. Concurrently, a strengthened subpolar gyre enhances the northward advection of saline subtropical waters, temporarily reinforcing the AMOC (fig. S13).

However, within a decade of the eruption (years 7706 to 7720), high-latitude cooling promotes extensive sea ice growth. The resulting summer seasonal melt freshens the upper ocean and establishes a persistent halocline. This stratification suppresses deep convection, initiating a sea ice–NADW feedback that drives a sustained AMOC weakening. Several decades later, the trajectories of the AMOC across the ensemble of simulations become increasingly governed by the driving forces and feedbacks associated with the internal variability of the coupled ocean-atmosphere system and are therefore heavily influenced by random perturbations. In the following, we explain this dynamical behavior using a stochastic modeling framework.

### Dynamical systems perspectives on postvolcanic decadal climate variability

While the physical, process-based mechanisms observed in the complex climate model help frame the initial interannual response of the climate system to volcanic forcing, the longer-term, multidecadal response remains less clear. The large ensembles, with and without volcanic forcing, show consistent behavior in the early stages after the eruption but quickly diverge on multidecadal timescales. Interpreting this long-term response through a process-based attribution for each individual ensemble member would require a complex, separate analysis. Instead, we approach this from a different perspective, one that allows for a more general physical understanding within the framework of nonlinear dynamical systems theory.

**Fig. 7.**
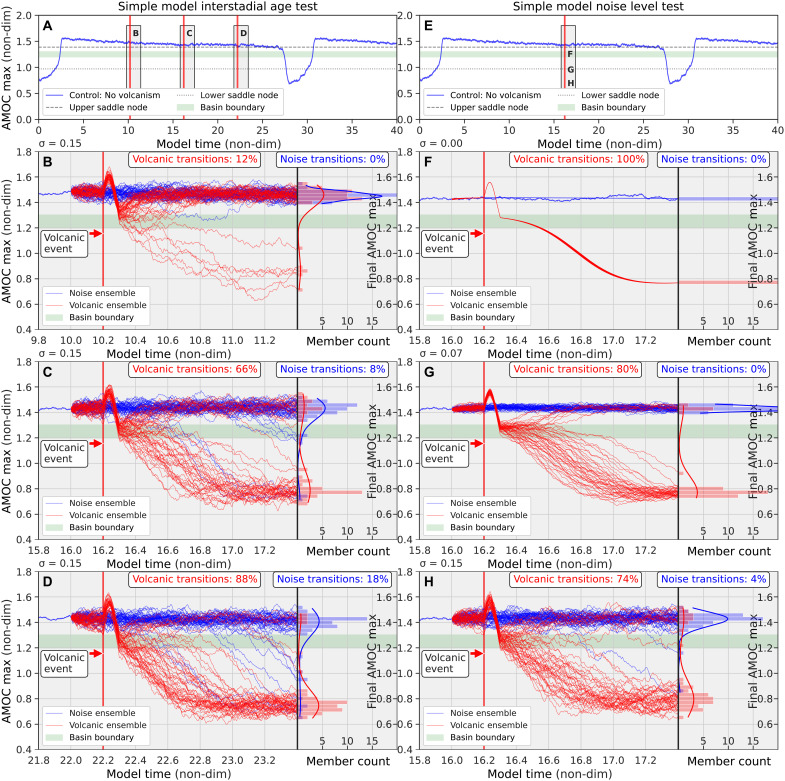
Interstadial transitions in a simple D-O model. A dynamical systems model with simulated volcanic eruptions is used to test the system response as the system moves closer to a tipping point (left), as well as a test of response to the amount of noise in the system (right). (**A**) A control simulation with no volcanism and with a fixed point that allows only for noise-induced transitions. The fold saddle node bifurcation points are indicated by dashed lines in (A) and the shaded green area indicates a basin boundary where the system flow changes vertical direction. (**B** to **D**) Simulated response of an ensemble of simulations with perturbed initial conditions to both volcanic (red) and noise-induced (blue) forcing of the simple model illustrated in (A). (**E**) The same as in (A) but with a time slice used for a noise sensitivity analysis. (**F** to **H**) Simulated response of the system with (red) and without volcanic eruptions (blue) under increasing noise levels (σ).

To better interpret the behavior of our complex climate model experiments, we use a simplified dynamical systems model (*19*) designed to mimic the qualitative dynamics of the full model (Fig. 7). The utility of this simple model lies in its ability to reproduce key features of the complex model by tuning its parameters. Specifically, we integrate a set of stochastic ordinary differential equations (SDEs) using the Euler-Maruyama method, incorporating a short-lived “volcanic” forcing (by simply integrating a cosine function in the SDE solution). After the forcing ends, the system evolves freely, simulating the postvolcanic AMOC response.

By adjusting the magnitude of internal noise, the amplitude of the volcanic perturbation, and the position of the fixed point in the dynamical system (Fig. 7, A to D), we are able to reproduce behavior qualitatively similar to that seen in the complex model (Fig. 3, A to D), although transition probabilities differ. The best agreement between the models occurs when the simple system is configured with a stable fixed point, which aligns with a strong stable AMOC. The behavior of the simple system is also found to be sensitive to the level of noise in the SDEs and has been adjusted in a heuristic manner to model the complex climate model. The tipping point behavior of the AMOC transitions in the complex model, consistent with the behavior of the simple model, suggests that these transitions are primarily noise-induced under the fixed background CO_2_ level used in the simulations. The large ensemble results from the complex climate model support the idea of a system with two quasi-stable states in AMOC strength, as previously discussed. From a dynamical systems perspective, such a system may either be stable with infrequent, noise-induced transitions, or it may reside in an unstable regime near a saddle-node bifurcation (a tipping point), where relaxation oscillations can occur.

**Fig. 8.**
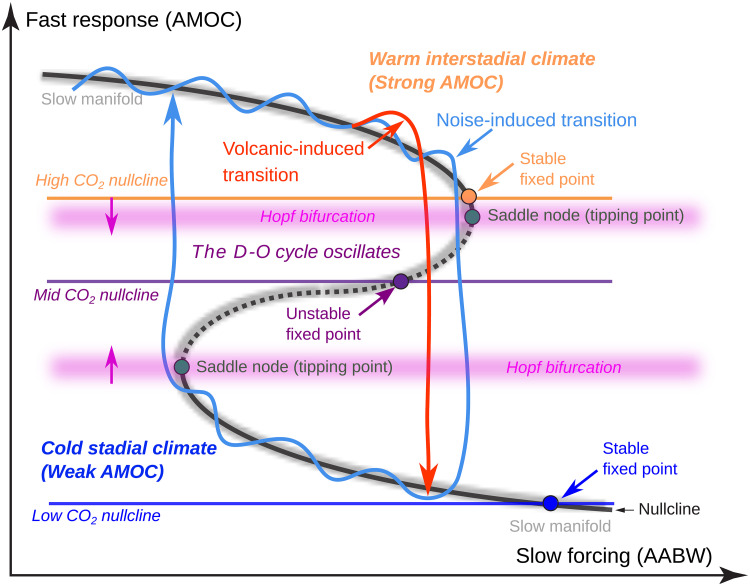
The D-O dynamical system. The proposed configuration of the underlying dynamics in the complex climate model at CO_2_ = 230 ppm with a high CO_2_ nullcline. At this high CO_2_, the system has a stable fixed point where a system without noise would stabilize to a fixed level of AMOC (orange fixed point). When the system control parameter (the CO_2_ nullcline) is lowered from high to mid (or raised from low to mid), the system goes through a Hopf bifurcation at the location of the saddle node and the system can then oscillate between strong and weak AMOC states (a D-O oscillator). In the complex climate simulations presented here, we propose that the climate model would remain in a fixed strong AMOC state in the absence of internal climate variability (noise). The atmospheric and ocean climate variability can create a noise-induced cycle between AMOC states (the light blue trajectory). A volcanic-induced transition (the red trajectory) can push the system through a tipping point prematurely and induce a transition to a lower AMOC state well before reaching the area of the saddle node tipping point (it would then recover back to the upper manifold). The internal variability can also inhibit the volcanic event from transitioning to the low AMOC state. The abscissa is the Southern Ocean forcing as in (*19*).

The CO_2_ concentration determines the position of the system’s fixed point. When the fixed point is close to a bifurcation and the system contains sufficient internal noise, it becomes difficult to distinguish whether the system is in an unstable (oscillatory) or stable (nonoscillatory) configuration, particularly since natural internal variability cannot be removed in the complex model. In the unstable regime (beyond a Hopf bifurcation), the AMOC should exhibit signs of critical slowing down or other early warning signals (*93*, *94*). However, such behavior has not yet been clearly demonstrated in these glacial climate simulations (see Fig. 8 for an illustration of these concepts).

Late in the interstadial phase, following the subsidence of a volcanic event, some realizations of the CCSM4 model shift into a colder state, while others fail to complete the transition from interstadial to stadial. Using the simple model presented here, and driven by Gaussian white noise to represent internal modes of natural variability, or wind stress fluctuations, reveals interesting dynamics: As the noise level increases, the system transitions from deterministic behavior (in the absence of noise, which cannot be simulated in CCSM4) to a regime where internal variability plays a dominant role (Fig. 7, F to H).

Figure 7F shows a large ensemble of simple model simulations with no noise but identical volcanic-like forcing applied to perturbed initial conditions. This tuned dynamical system has a control parameter (representing background atmospheric CO_2_) that places the system in a stable, nonoscillatory state with a fixed point just above the tipping point (the upper saddle node in Fig. 8). In this configuration, no transitions would occur in the absence of noise (blue ensemble in Fig. 7F). This corresponds to a state before a Hopf bifurcation would occur as the CO_2_ control parameter is lowered past the saddle-node threshold.

The sinusoidal-like postvolcanic AMOC response (red ensemble), similar to that seen in the complex model, forces the system across the basin boundary (green-shaded area), transitioning it to the lower manifold (cold, low AMOC state), from which it eventually recovers to the warm state, since the upper manifold contains a stable fixed point or attractor (see Fig. 8). As noise is gradually introduced into the system (Fig. 7G), most of the volcanic ensemble (red) still transitions. However, with higher noise levels (Fig. 7, H and C), the volcanic perturbations become less effective at inducing transitions, and the system increasingly remains in the upper manifold (warm, high AMOC state). There is some evidence of this resilience to tipping in the behavior of the complex model (Fig. 3, C and D). Overall, the simple dynamical systems model captures key aspects of the complex climate model’s behavior and provides an additional lens through which to understand the underlying dynamics of the modeled glacial climate system.

## DISCUSSION

This study presents a hypothesis that some of the large and rapid climate fluctuations observed on millennial timescales within the MIS 3 ice core record could result from intermittent volcanic activity. Background conditions such as Earth’s orbital configuration, greenhouse gas concentrations, and ice sheet size, determine whether the glacial climate system resides in a favorable “sweet spot” conducive to D-O events (*21*). When the climate system lies well outside this range, as during the Holocene, it may exhibit greater resilience to isolated, large volcanic eruptions. However, this hypothesis requires testing through probabilistic modeling.

The absence of very large abrupt climate changes during the early Holocene despite several major eruptions releasing more than 230 Tg of sulfate between 10 and 5 ka, supports this idea. A similar climate resilience outside the D-O sweet spot may have existed earlier in the glacial period, when extremely large volcanic eruptions occurred during or were followed by relatively long interstadial periods. This appears to be the case during MIS 5a around 80 ka, although this remains a topic of debate (*37*, *50*, *95*, *96*).

During MIS 5a, relative sea level was approximately 30 to 40 m higher than during MIS 3, indicating much reduced continental ice sheets (*97*). Atmospheric CO_2_ concentrations and annual average Greenland surface temperatures were likely higher by approximately 10 to 15 ppm and 5°C, respectively, compared to peak interstadial conditions during MIS 3 (*98*, *99*).

A more promising research direction would be to investigate the role of clustered volcanic eruptions, rather than isolated extreme events, in driving millennial-scale climate variability during the last glacial period (*74*, *100*). Prolonged periods of persistent volcanic activity, even if individual eruptions are of lower volcanic explosivity index, might increase internal climate variability and potentially produce effects similar to, or event counteracting, those modeled in this study.

Our findings suggest that increased internal climate variability, or noise, could enhance the glacial climate system’s resilience to abrupt AMOC collapse and may have implications for the modern climate as studies have shown that anthropogenic warming leads to increased climate variability (*101*, *102*). Recent studies indicate a high likelihood that the AMOC will transition to a shutdown state within this century (*9*, *103*), and our study provides a perspective on the timescale of this shutdown, taking approximately two centuries to complete. However, differences may exist between modeling studies applying artificial forcing to induce an AMOC slowdown and those capturing naturally occurring transitions, such as those modeled here for the glacial period. Furthermore, the stability characteristics of the AMOC may differ between modern and glacial climates (*104*). Regardless of these epochal differences, even an initial slowdown in AMOC circulation under climate change this century has important implications for humanity, as projected by models participating in the CMIP phase 6 (CMIP6) (*105*), and therefore requires further investigation in the context of past climate change.

## MATERIALS AND METHODS

The model used in this study is the CCSM4, configured at approximately 3° × 3° spatial resolution in both the atmosphere and ocean (*80*). The atmospheric component, CAM4, includes 26 vertical levels extending from the Earth’s surface to the top of the stratosphere (3.5 hPa or 40 km). This model was used in previous CMIP5 simulations and has been widely used to simulate D-O–like behavior under glacial boundary conditions (*16*, *19*, *106*).

The AMOC variability in CCSM4 has been extensively documented for both the standard-resolution (*107*) and low-resolution configurations (*80*). While the low-resolution model used in this study retains key features of the AMOC, it exhibits mixed layer depths that are shallower than those simulated in the standard-resolution version. In our modified version of CCSM4, a bathymetry-dependent vertical mixing scheme (*108*) has been replaced by a generic (*109*) enhanced mixing profile below 2500 m. The change was made because of uncertainties in reconstructing past tidal mixing during glacial periods (*110*) (see Supplementary Text).

To validate this model configuration, we compare it against 20th-century observations using an ensemble of six CCSM4 simulations forced with historical 20th-century conditions. We find that the model captures greenhouse gas and volcanic forcing responses realistically and that the simulated 20th-century warming aligns well with observed temperature trends (fig. S4). Discrepancies mainly arise in the phase of the ENSO, and volcanic events in the model produce a slight cold bias compared to observations (see Supplementary Text). The mean modern AMOC strength in this model is reasonably well represented at approximately 15 Sv. Further details regarding the implementation and parameterization of volcanic forcing in CCSM4 are provided in the Supplementary Text.

A large language model (LLM) was used to help improve the flow, readability, spelling, and grammar of some of the text paragraphs here. The LLM used was Qwen3-235B-A22B (https://huggingface.co/Qwen/Qwen3-235B-A22B, https://github.com/QwenLM/Qwen), which falls under the Apache 2.0 license. The LLM was not used for any scientific hypotheses or inductive reasoning in deriving any of the scientific content here. No research was done with the LLM. The prompt provided to the LLM to improve selected sections of text from the paper was as follows: “In the following text, please help improve the flow, grammar, spelling, and readability of the paragraph, do not change any of the content.”

## Supplementary Materials

### This PDF file includes:

Supplementary Text Figs. S1 to S13 References
