# Supplementary material for: Volcanism-induced collapse and recovery of the Atlantic meridional overturning circulation under glacial conditions
Source: Sci Adv. 2026 Feb 4;12(6):eadx2124. doi: 10.1126/sciadv.adx2124 (PMC12871447; doi:10.1126/sciadv.adx2124)
Supplement: Supplementary file 1 — Supplementary Text Figs. S1 to S13 References [file sciadv.adx2124_sm.pdf]

Science Advances
AAAS

Supplementary Materials for

Volcanism-induced collapse and recovery of the Atlantic meridional overturning circulation under glacial conditions

Guido Vettoretti *et al.*

Corresponding author: Guido Vettoretti, 2guido@gmail.com

Sci. Adv. **12**, eadx2124 (2026)
DOI: 10.1126/sciadv.adx2124

This PDF file includes:

Supplementary Text
Figs. S1 to S13
References

Supplementary Text

Volcanic Extreme Value Analysis

GEV Distributions

The Generalized Extreme Value (GEV) distribution is a family of continuous probability distributions used to model the maximum (or minimum) of a number of samples of various distributions (*112*). It encompasses three types of extreme value distributions: Gumbel, Fréchet, and Weibull, making it versatile for different types of data. In the application in this paper we are interested in a positive definite probability distribution with a fat tail that spans to the right. The cumulative distribution function (CDF) of the GEV distribution is

$$F(x;\mu,\sigma,\xi) = \exp\left\{-\left[1+\xi\left(\frac{x-\mu}{\sigma}\right)\right]^{-1/\xi}\right\} \tag{1}$$

where three parameters, ξ, μ and σ represent the shape, location, and scale of the distribution function, respectively. Note that σ and $1 + \xi(x-\mu)/\sigma$ must be greater than zero. The shape and location parameter can take on any real value. The resulting probability distribution function (PDF) for two category of shape parameter (i.e., whether it is equal to zero or not) is

$$P(x;\mu,\sigma,\xi) = \frac{1}{\sigma} t(x)^{\xi+1} e^{-t(x)} \tag{2}$$

where

$$t(x) = \begin{cases} \left[1+\xi\left(\frac{x-\mu}{\sigma}\right)\right]^{-1/\xi} & \text{if } \xi \neq 0 \\ e^{-(x-\mu)/\sigma} & \text{if } \xi = 0 \end{cases} \tag{3}$$

The shape parameter (ξ) governs the tail behavior of the distribution:

- When $\xi > 0$, the distribution displays a heavy tail, resembling the Fréchet distribution. This is useful for modelling extreme values with potential for larger maxima. We use $\xi > 0$ for modelling the volcanic event size distribution.
- For $\xi = 0$, the distribution takes the form of Gumbel, characterized by exponential-type tails.
- In the case of $\xi < 0$, the distribution exhibits a bounded upper limit, resembling the Weibull distribution, which is suitable for modeling data that does not exceed a certain threshold.

The location parameter (μ) shifts the distribution along the x-axis, indicating the center of the distribution and influencing the mean and median of the data modeled by the GEV distribution. Adjusting μ aligns the

distribution with the observed data. The scale parameter (σ) either stretches or compresses the distribution. A larger σ leads to a wider distribution, signifying more variability in extreme values, while a smaller σ concentrates the distribution around the location parameter.

Return Level Plots

Estimates of extreme quantiles of the annual maximum distribution are then obtained by inverting the GEV cumulative distribution function (*113*):

$$z_p = \begin{cases} \mu - \frac{\sigma}{\xi}\left[1 - \{-\log(1-p)\}^{-\xi}\right], & \text{for } \xi \neq 0 \\ \mu - \sigma \log\{-\log(1-p)\}, & \text{for } \xi = 0 \end{cases} \tag{4}$$

where $G(z_p) = 1 - p$. Here, z_p is the return level associated with the return period $1/p$, since to a reasonable degree of accuracy, the level z_p is expected to be exceeded by the annual maximum in any particular year with probability p. Since quantiles enable probability models to be expressed on the scale of data, the relationship of the GEV model to its parameters is most easily interpreted in terms of the quantile expressions above. In particular, defining $y_p = -\log(1-p)$, so that

$$z_p = \begin{cases} \mu - \frac{\sigma}{\xi}\left[1 - y_p^{-\xi}\right], & \text{for } \xi \neq 0 \\ \mu - \sigma \log\left(y_p\right), & \text{for } \xi = 0 \end{cases} \tag{5}$$

it follows that, if z_p is plotted against y_p on a logarithmic scale - or equivalently, if z_p is plotted against $\log\left(y_p\right)$ - the plot is linear in the case $\xi = 0$. If $\xi < 0$ the plot is convex with asymptotic limit as $p \longrightarrow 0$ at $\mu - \sigma/\xi$; if $\xi > 0$ the plot is concave and has no finite bound.

The volcanic timeseries for the Holocene and MIS3 are not continuous and therefore the return level analysis is not straightforward. For example the data is written skipping several years or centuries in the respective timeseries. Return level analyses use the extreme values from a continuous time step. For example, to understand climatological extreme precipitation values, one would take the annual maximum of precipitation during each annual cycle and make a timeseries. The return plot is then much simpler to determine as the timestep is 1 year. The volcanic return time analysis requires binning the timeseries and taking the maximum in a specific interval. We have chosen the smallest interval of time in each dataset (Holocene and MIS3) where there are no missing values in the selected interval. The binning length for the Holocene (from -10000 to 2000 years CE) data is 47 years long and the MIS3 (57 ka to 27 ka) binning length is 1521 years.

CCSM4 Model and Volcanic Forcing

The Glacial CCSM4 Model and Boundary Conditions

We use the National Center for Atmospheric Research (NCAR) Community Climate Model Version 4 (CCSM4) at 3x3 degrees resolution in the atmosphere and ocean. There are 26 vertical levels and the model top is at 3.5 hPa (approximately 40 km). The model includes sea ice and land surface, but is run without ocean biogeochemisty. The land surface has a simple carbon-nitrogen cycle. For paleo-climatic boundary conditions, the model uses fixed Last Glacial Maximum (LGM) ice sheets and orbital insolation. The model has been used in several studies to investigate the mechanisms responsible for D-O climate variability. Recently, the model has been used to show the appearance of D-O variability in a window of background atmospheric CO_2 concentration (*19*). CCSM4 has been used to accurately replicate the last glacial cycle climate variability (*16, 22, 106, 114*). The simulations demonstrate high amplitude near millennial-scale variability in the AMOC and corresponding temperature changes at Greenland and Antarctica that are in agreement with observed proxy climate records (*8, 99, 115*). The model differs from the original NCAR CCSM4 in that changes are made to the tidal mixing parameterizations used to infer ocean diapycnal diffusivity (*108*). In (*16, 106*) a fixed background profile of Bryan and Lewis type (*109*) (BL79) mixing with a transition depth of 1000 meters was used in this higher resolution simulation. The tidal mixing and overflow parameterizations were turned off. Here we do the same in the low resolution version but instead use a transition depth of 2500 meters for the BL79 vertical mixing profile for the 3x3 degree version of the model. This modification was made because the ice age turbulent kinetic energy dissipation by tide/bathymetry interaction and the ice age bathymetric overflows are not well constrained. Studies using tide amplitude models have suggested that the internal wave mixing energy during the LGM was approximately 3 times the modern values (*110*), however, it is not clear how this will impact the climate during MIS3 when much of the D-O variability is present and ice sheets are constrained to be different from LGM (*116*). Other studies indicate that glacial atmospheric climate forcing may be as important in setting the stratification of the LGM ocean (*117*).

Volcanic forcing used in CCSM4

In the standard CCSM4 model, stratospheric volcanic aerosols omit zonal variations in stratospheric mass loading (Fig. S1). The volcanic input consists of monthly-mean masses (kg m^{-2}) distributed on a predefined meridional and vertical grid (*118*). We inject 58, 115, and 230 Tg of volcanic aerosols representing large to very large volcanic eruptions such as 1991 Pinatubo, 1815 Tambora, and 1257 Samalas (*76–78*). This mass

represents the total aerosol content, comprised of 75% sulfuric acid (H_2SO_4) and 25% of water (H_2O). The eruption month is set to January, which is the default for past volcanic forcing when then eruption month is unknown (*49, 119*). In CCSM4/CESM1(CAM4), stratospheric aerosols from 1850 to 2010 are prescribed based on (*118*) and following (*76*). The aerosols are assumed to have a constant log-normal size distribution, with a wet effective radius (reff) of 0.426 μm and a standard deviation (σ(ln r)) of 1.8, as noted in (*120*) (see also Table 1 from (*120*)).

The supplementary materials in Bethke et al. (*10*) highlights potential caveats related to large volcanic events. In the CAM4 microphysics model, aerosols do not influence cloud drop sizes, as the CAM4 physics package employs prescribed bulk aerosols, the older CAMRT radiation package (not RRTMG), and Rasch-Kristjánsson (RK) microphysics. This configuration includes only direct aerosol effects, with no indirect or semi-direct effects. Another way aerosols affect climate is through the deposition of dust and carbon species, which alter surface albedo. In CAM4, when running with prescribed aerosols (the default setting), aerosol deposition is read from datasets by both the CLM and CICE components.

The spatio-temporal shape functions of volcanic forcing are shown in Fig. S1a for latitude versus month after the eruption. Also shown is the height-latitude shape function (Fig. S1b), which follows the Brewer-Dobson circulation and is used in typical attribution studies of 20th century climate run using CCSM4 (*118*). In Fig. S1d the amplitude of the Volcanic AOD in the model is larger by approximately 0.1 throughout the 20th century. This will tend to overestimate the cooling in the model. In the main manuscript, the glacial climate simulations that are forced with large volcanic eruptions have AOD that are fixed and do not change depending on the climate state. The stadials and interstadial in the glacial climate simulations have the same AOD forcing when a fixed sulfate load is prescribed, as the forcing is not interactive. Fig. S1e, f, g shows the model global mean AOD in the Equatorial volcanic forcing for the 230, 115 and 58 Tg volcanic aerosol experiments.

The Vertical Shape Function

The vertical profile of the shape function Fig. S1c that was used in the paleoclimate D-O simulations was different than that used in the modern 20th century NCAR simulations S1b. Fig. S1e shows that the global mean AOD response to a tropical eruption with a 500 Tg (paleo shape function) sulfate load in the simulations used in this study equates to the response of a 230 Tg volcanic aerosol load, with a slightly steeper drop after reaching peak AOD in the paleo simulations. Using a paleo vertical shape function of 250 and 125 Tg of sulfate aerosol load corresponds to an equivalent load of 115 and 58 Tg volcanic aerosol using the 20th century shape function, respectively. We find this volcanic parameterization used in CCSM4 is fairly

linear in the response between mass loading and AOD. The stratospheric sulfate mass loading is converted to an AOD forcing during model runtime and saved in the model output, therefore we are able to precisely compare the forcing exerted by the different vertical shape functions. While the latitudinal distribution of AOD in the low latitude regions equatorward of 30°N/S is unchanged, the paleo profile causes some modest AOD increase in the 30-45° mid-latitude band and more substantial AOD decrease poleward of 60° in both hemispheres, with the global means being equivalent (Fig. S2). Our main findings are robust to the details of the latitudinal variations in the two different spatial AOD forcing distributions. To test this, we reran one of the ensemble of volcanic simulations at year 7701 to assess the differences in vertical shape function (Fig. S3). The entire set of 6 ensembles were extremely compute intensive, so we did not repeat this procedure for the other ensembles. The differences in volcanic induced transition probabilities was 72% versus 78% for the volcanic event starting at year 7701.

The nature of the prescribed volcanic forcing originally developed in CCSM4 is not without problems. The lack of transport of sulfate aerosols to interhemispheric regions during large volcanic events adds to the uncertainty of the results presented here. The scaling of sulfate deposition to radiative forcing does not adequately account for nonlinearities in aerosol formation and op tical properties. The assumption of reconstructions using fixed particle sizes where actual sizes may vary widely is detrimental, as well as the lack of complex aerosol chemistry that converts SO_2 to sulfate aerosols. Simplifications in the aerosol removal process can lead to unrealistic durations of volcanic forcing in the model. These simplification can lead to an overestimation of radiative cooling compared with climate proxies. The prescribed distribution of volcanic aerosols does not capture the diverse spatial or temporal variability observed in actual eruptions (*79, 121*). Inaccuracies in ice core dating can also create a misalignment between volcanic events and the climate response, affecting the validity of the forcing data (*49, 53, 122*).

Volcanic Variability: Modern Era Validation

To determine the sensitivity of the millennial-scale climate to volcanism using this paleoclimate version of CCSM4, we investigated the model's ability to represent the observed 20th century climate record and the attributed influence of volcanism. Fig. S4 shows the temperature response in simulations of 20th century climate using CCSM4. The 20th century simulations presented here use the same ocean mixing modification used to obtain accurate representations of the D-O oscillation. An ensemble of 6 simulations was used to ascertain the natural climate variability in the model from 1850 to 2000. Surface air temperature anomalies are plotted versus the mean of the 1960s to 1980s. The model ensemble mean of six simulations using a 20th

century run is compared against GISS observations (*123,124*) and NCEP reanalysis (*125*) of global surface air temperature. Surface air temperature from both the observation based data and model ensemble demonstrate good agreement. The volcanic SO_2 load from major volcanic eruptions during the 20th century are used as forcing (*118*). For the period 1883-1990, aerosol optical depths are estimated from optical extinction data, whose quality increases with time over that period. For the period 1850-1882, aerosol optical depths are more crudely estimated from vulcanological evidence for the volume of ejecta from major known volcanoes. All other forcings are also included in the model simulations (e.g. solar insolation, agricultural changes, CO_2 and other greenhouse gases etc.). The volcanic forcing through the 20th century is strongly correlated with the cold negative anomalies seen in both the model ensemble and the observations. The rate of change in the temperature through the 20th century is in good agreement with the model ensemble. However, the model tends to be too cold due to the higher modelled AOD than observed (*126*) (Fig. S1d). Variations also occur because of the differences in ENSO phase and the model fidelity in capturing the ENSO oscillation (*80*). However, there is and overall good agreement between the temperature anomaly change from 1900 to 2000 in the observations compared with the model, which demonstrates the fidelity of the model in capturing the 20th century climate.

The relationship between ice-core sulfate concentrations and stratospheric sulfate loading is uncertain for eruptions larger than Pinatubo (1991) and more uncertain for very large eruptions (e.g., Tambora 1815, Samalas 1257) (*10*). Aerosol microphysics suggests that larger SO_2 injections produce larger aerosol sizes with smaller radiative effects per unit mass of sulfur (*127*). Thus, the linear scaling (*121*) may overestimate the radiative effects of the largest eruptions in our model because the radiative effects are smaller for increased aerosol sizes (*127*). Crowley et al. (*122*) suggested a 2/3 power scaling for eruptions larger than Mt. Pina tubo to mitigate these effects, something that should be considered in future applications. While further research is required to better constrain the forcing of very large eruptions, our ice-core based volcanic forcing is to first-order proportional to relevant surface temperature reconstructions (*53,128*). Previous studies have questioned whether the climate response to volcanic forcing is state-dependent (e.g. Glacial or Holocene) (*129,130*). In our glacial volcanic study we may overestimate the effects of the largest volcanic eruptions. We have run a sensitivity study to see if there are differences in volcanic eruption climate response to very large magnitude eruptions (230 Tg of volcanic aerosol) such as 1257 Samalas (*77, 78*) in both the modern day and glacial simulations. We find a general cooling of between 2.5 to 3.0 $^{\circ}C$ cooling with the glacial stadial period showing the strongest response. The top of the atmosphere radiation imbalance (TOA) shows a large, greater than 15 W m^{-2} global average radiation imbalance one year following the volcanic eruption (Fig. S5). These responses are in the bulk of other climate model simulations reflecting the large uncertainty of aerosol size

for very large volcanic eruptions (*83, 127, 131*).

Additional Supporting Model Diagnostics

Surface Temperature and Mean Sea Level Pressure anomalies

The large equatorial volcanic eruptions lead to general cooling throughout the planet, especially over the continental masses. Here we use the anomaly to represent the differences between the period after the volcanic event versus the climatology before the event. There is a particularly strong cooling in the Northern Hemisphere over North America and over Western Europe in the first year after the eruption. There is a warming anomaly in the high North Atlantic Ocean south of Greenland in regions of substantial deep water formation. There are warming anomalies in the Pacific and Indian sector of the Southern Ocean. There is an El Nino pattern superimposed on the strong cooling in the equatorial Pacific. Also there is a teleconnection between the Aleutian and Icelandic low areas (Fig. S6).

The mean sea level pressure also shows a similar response as observed in the surface temperature anomalies. Here there is a strong connection between the Pacific and Atlantic regions. There are large low-pressure anomalies over both mid-latitudes of the Eastern Pacific Ocean as well Eastern Atlantic Northern mid-latitudes after the volcanic eruption. There are large-scale high-pressure systems in the Arctic and over the Southern Ocean as well as over Antarctica. The Icelandic low and the Azores high weaken initiating an easterly surface flow anomaly over the high North Atlantic Ocean (Fig. S7).

While a number of studies have demonstrated a positive NAO response occurring after a volcanic event (*132*) the composite NAO index of the ensemble mean 20th Century simulations (Fig. S4) shows some correspondence to a positive NAO but not precisely in the following winter except for the Mt. Pinatubo eruption (Fig. S8). The NAO index is quite variable in the ensemble mean and it would be difficult to attribute much statistical significance to the correlation between the positive NAO index and the occurrence of the volcanic eruption in this set of modern simulations.

The monthly mean response the Nino 3.4 index and the NAO index both show similar patterns with large-scale anomalies occurring within the first and second years after the eruption. The majority of the changes are felt in the winter of the ensuing years (Fig. S9).

The DJF NAO structure using an Empirical Orthogonal Function (EOF) decomposition of a modern control run (e.g. shown in Fig. S4) and the D-O control run shown in the main manuscript (without volcanism) are markedly different (Fig. S10). Although they posses the characteristic NAO pattern in the first mode of variability, the structure of the mode is enhanced in glacial climate experiments, with a weaker low

pressure center over Greenland and a stronger high pressure center of the Azores. The Azores portion of the variability is much larger in extent, expanding more towards the North American continent. The second mode of variability explaining approximately a quarter of the variance in the modern and glacial simulations shows marked differences suggesting that the warm interstadial and cold stadial periods have modes of variability that are different than what the modern pattern presents. We should not expect that the glacial period NAO will respond in a similar manner in the winter season following a large volcanic eruption. In these results we have only shown the response to a large eruption after an interstadial eruption, so understanding the connections between a post-volcanic NAO and the modern or glacial climate requires investigations beyond this present study. The analysis presented in the main manuscript described only the preconditioning of the collapse of the AMOC to a volcanic event during the late interstadial period.

The winter NAO Index pattern in Fig. S11 is consistent with the timeseries in Fig. S9. The winter NAO Index weakens immediately after the volcanic event. The first Empirical Orthogonal Function (EOF) explains 58% of the variance and shows the typical pattern associated with the NAO. The second EOF mode captures 21% of the variance and also corresponds to a weakening of the NAO seen in the first EOF.

The time series of the anomalies over the subpolar gyre show a general pattern of the tipping of the AMOC gradually into the lower state over the 300 years after the volcanic event (Fig. S12). These trends are consistent with the initial response of the AMOC after the volcanic event described in the main manuscript.

The heat and salt transport anomalies between the volcanic experiment and no-volcanic control ensemble means observed over the 440 year period after the volcanic eruption is also consistent with changes in the AMOC (Fig. S13).

Supplementary Figures

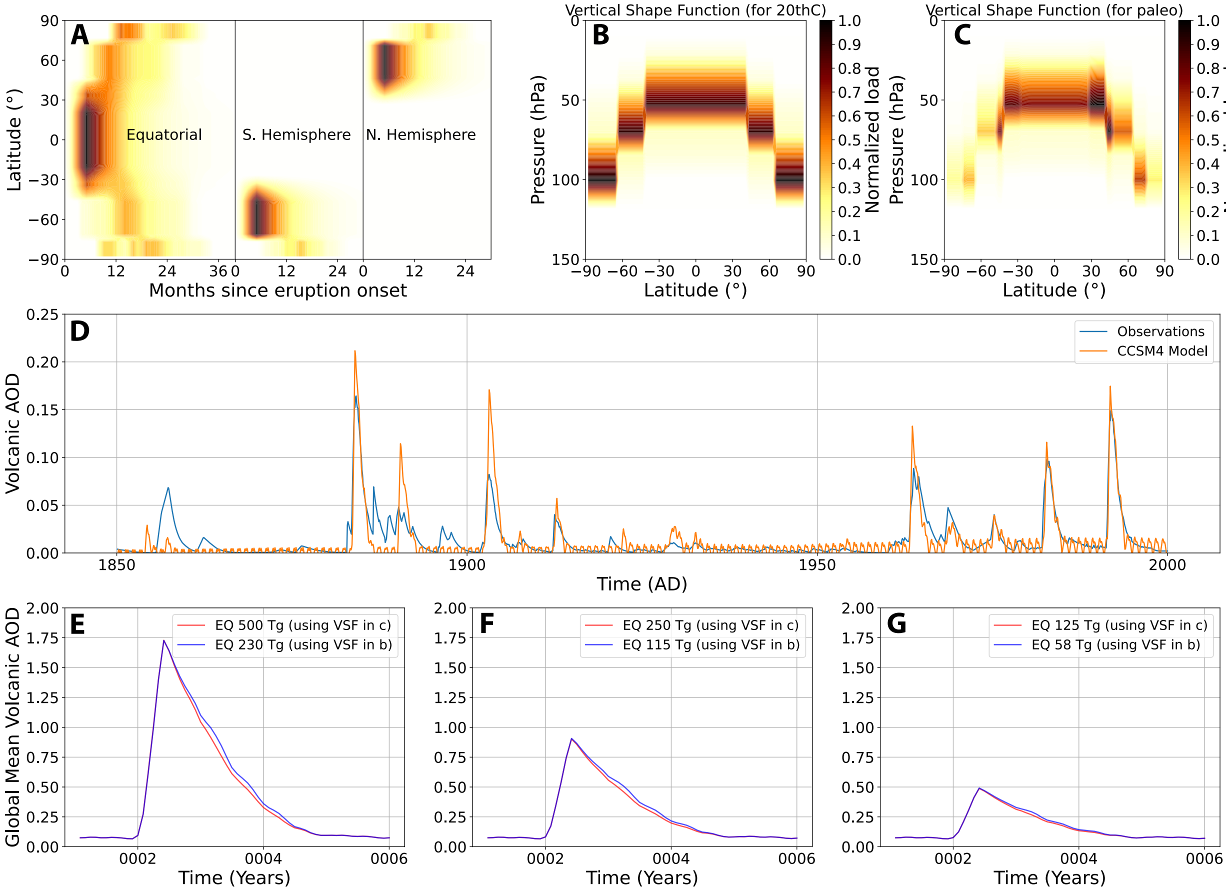

Figure S1: **CCSM4 mass mixing ratios and aerosol optical depth.** (**A**) Stratospheric layer aerosol mass mixing ratio in CCSM4 (kg kg^{-1} air normalized) from (*10*). (**B**) The ”20th Century” vertical shape function used in the simulation shown in (D) and the (**C**) ”paleo” vertical shape function used in this study. (**D**) Global mean aerosol optical depth (AOD) form a 20th century simulation in the CCSM4 model versus observations for this time period (*126*). Global mean AOD comparing simulations using the shape functions from either (B) (blue, ”20th Century”) or (C) (red, ”paleo”, this study) for (**E**) 230; (**F**) 115 and (**G**) 58 Tg of volcanic aerosol.

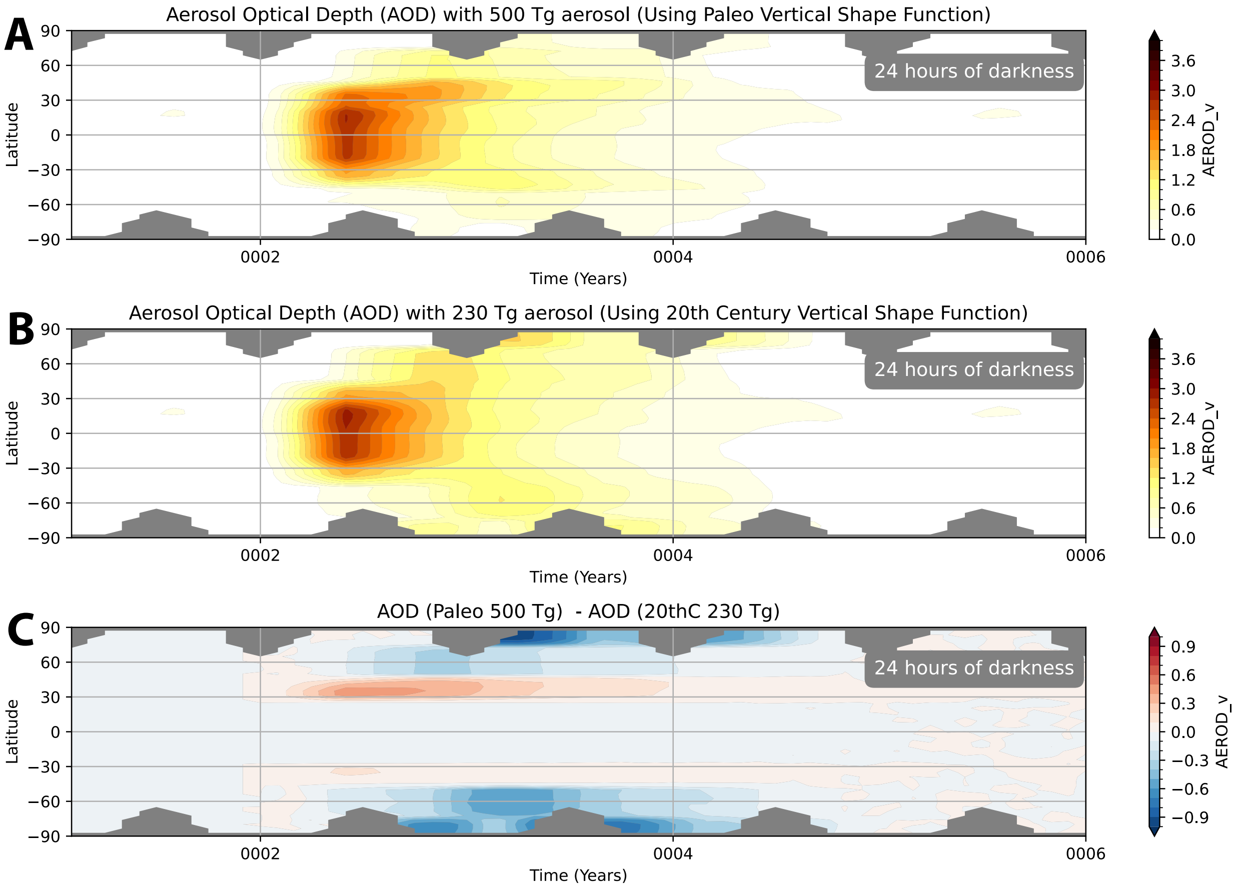

Figure S2: **Hovmöller diagrams of model aerosol optical depth.** AOD (dimensionless) for the (**A**) Equatorial 500 Tg volcanic aerosol (used in the paleoclimate experiments) and (**B**) 230 Tg volcanic aerosol (used in the original model for 20th Century runs) CCSM4 experiments illustrating how the (**C**) differences in the "paleo" vertical shape function produces a bias at mid to high latitudes after an equatorial volcanic event. The volcanic event forcing peaks in Boreal Summer 6 months after the eruption. The volcanic eruption occurs at Jan 1st of Year 2.

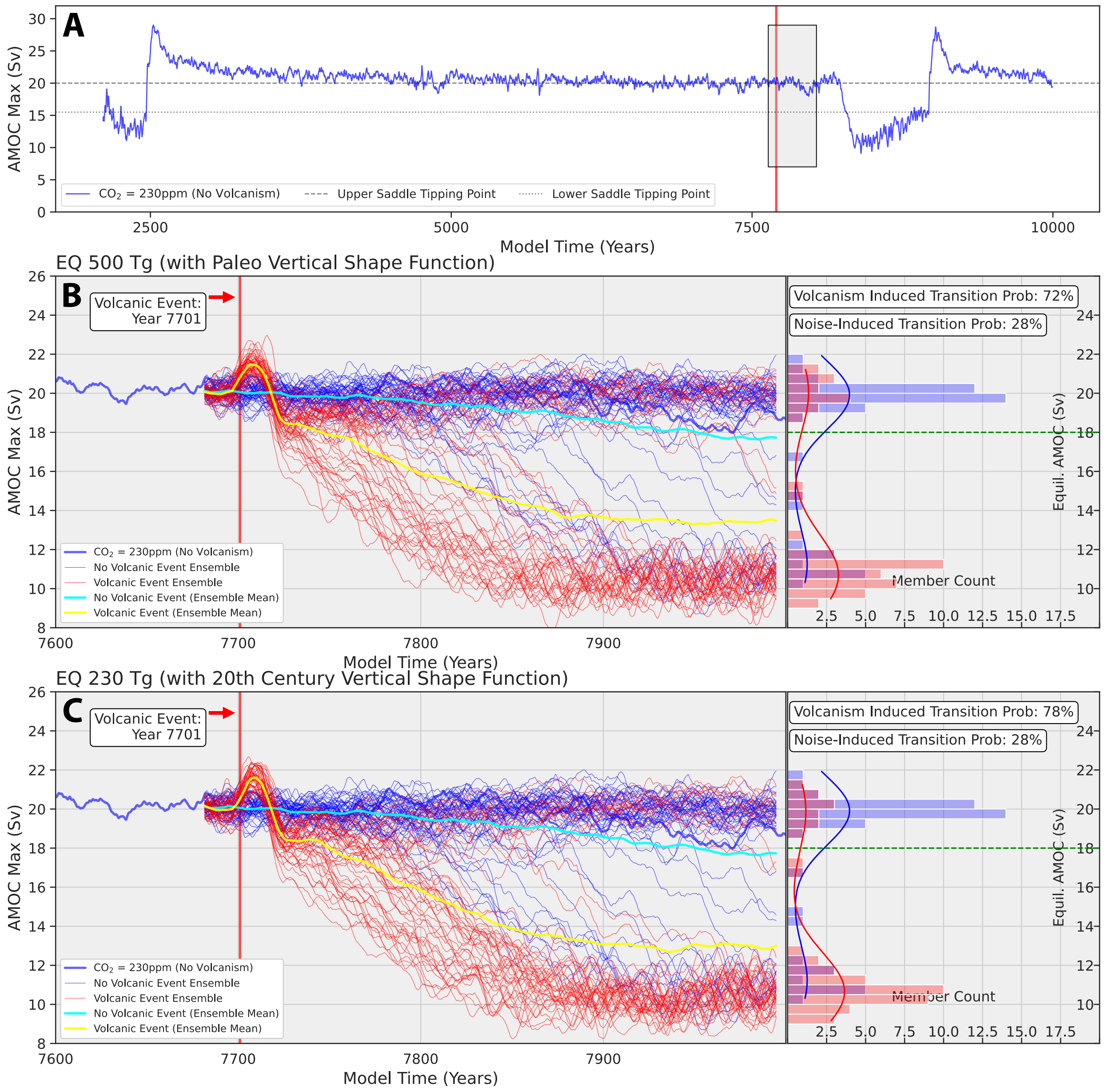

Figure S3: **Shape function impact on volcanic transition probabilities.** (**A**) The grey box shows the temporal location of the start of the volcanic perturbation experiment. (**B**) The 50 ensemble member volcanic mass loading 500 Tg aerosol experiment (used with the paleo vertical shape profile) and the (**C**) 50 member ensemble with the 20th Century vertical profile that corresponds with a sulfate aerosol load of 230 Tg (for the Equatorial eruptions). Volcanic experiments are in red and the noise internal variability experiments are in blue.

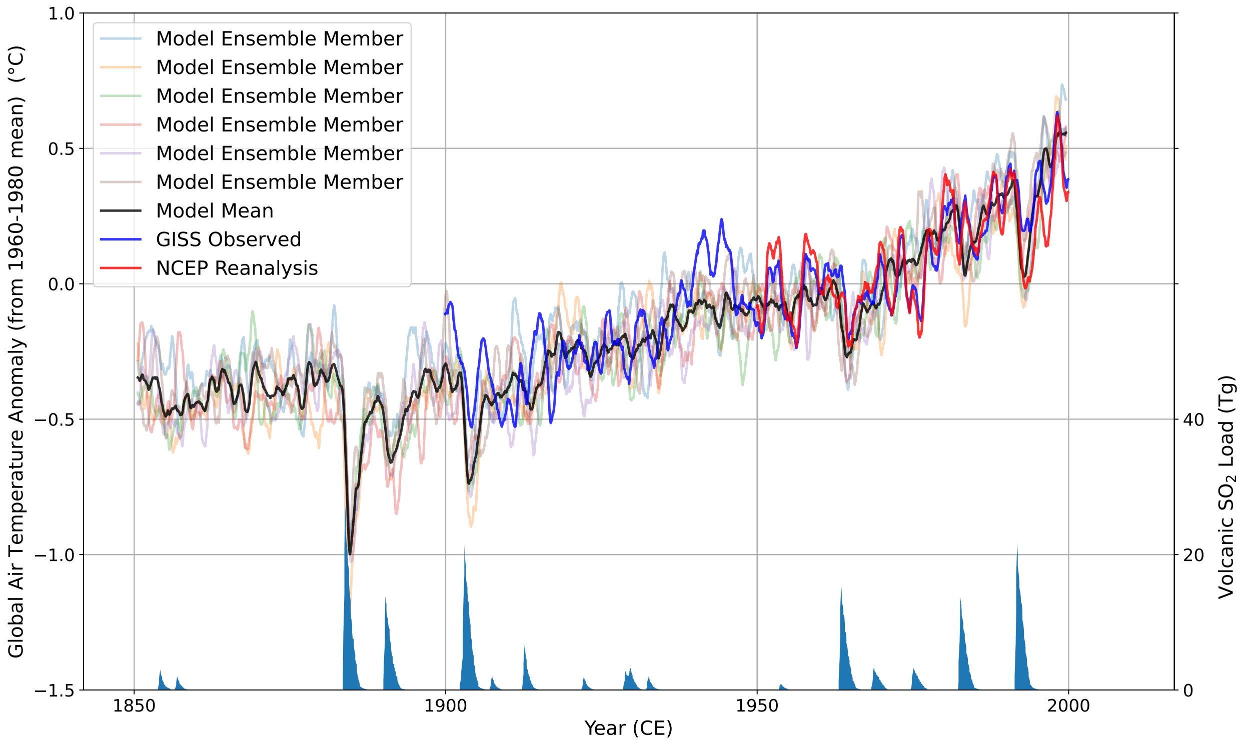

Figure S4: **Low-resolution CCSM4 modern 20th century climate variability.** Modern CCSM4 simulations under 20th century forcing using the ”D-O model” ocean mixing. The volcanic stratospheric SO_2 load is calculated from the aerosol forcing dataset used in CCSM4, which uses the 20th Century vertical shape function in Fig. S1B.

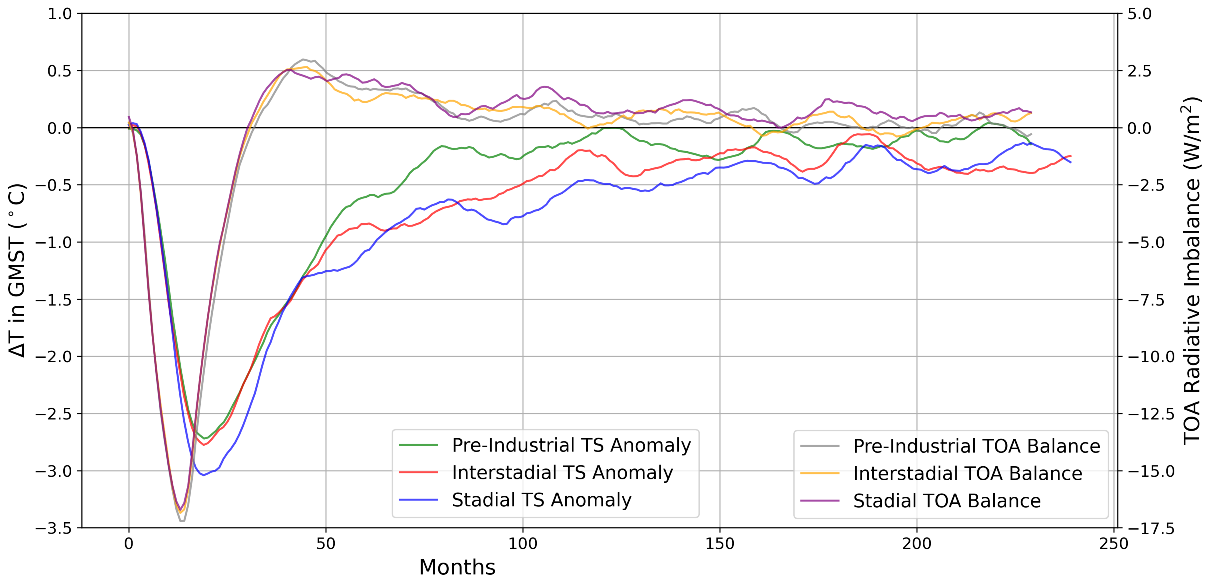

Figure S5: **Post-volcanic top of the atmosphere radiative response and global surface cooling.** The response to an equatorial 230 Tg volcanic aerosol eruption over a 20 year period for modern boundary conditions and glacial conditions during stadial and interstadial periods. The top of the atmosphere radiation imbalance (TOA) is also shown (right y-axis) so that we can see the lead and lags between the temperature response (left y-axis) at the surface.

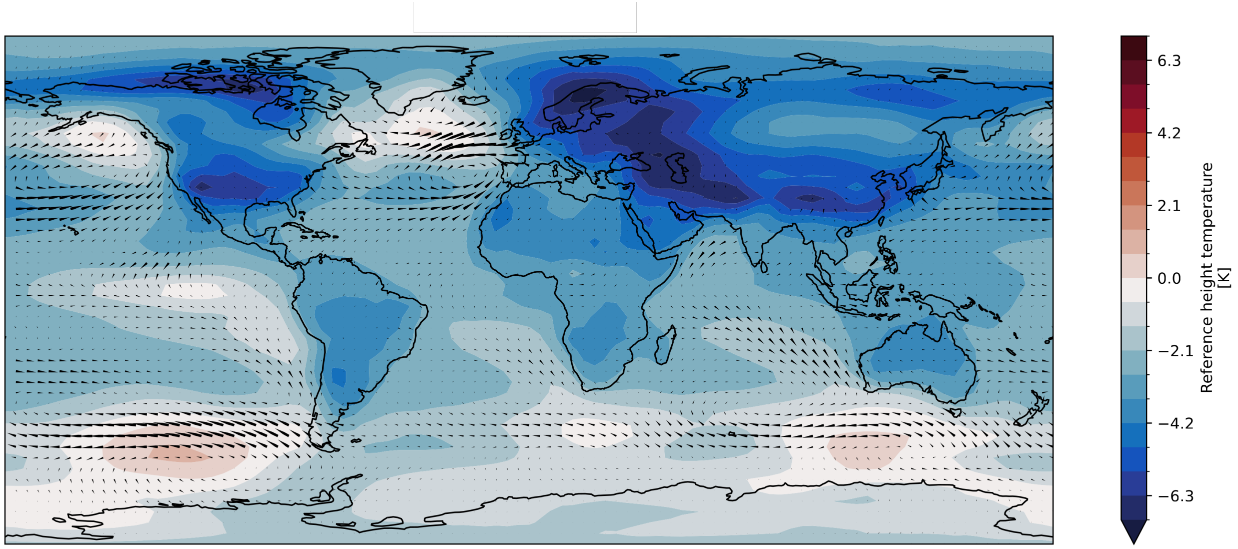

Figure S6: **Post-volcanic global surface temperature response.** The surface temperature response of the glacial climate system one year after an equatorial 230 Tg volcanic aerosol eruption. Plotted as an anomaly relative to the mean climatology before the event (years -25 to 5 averaged). There is an El Nino anomaly in the Pacific relative to the surroundings but is negative because the anomaly is compared to the climatology in the period previous to the volcanic event.

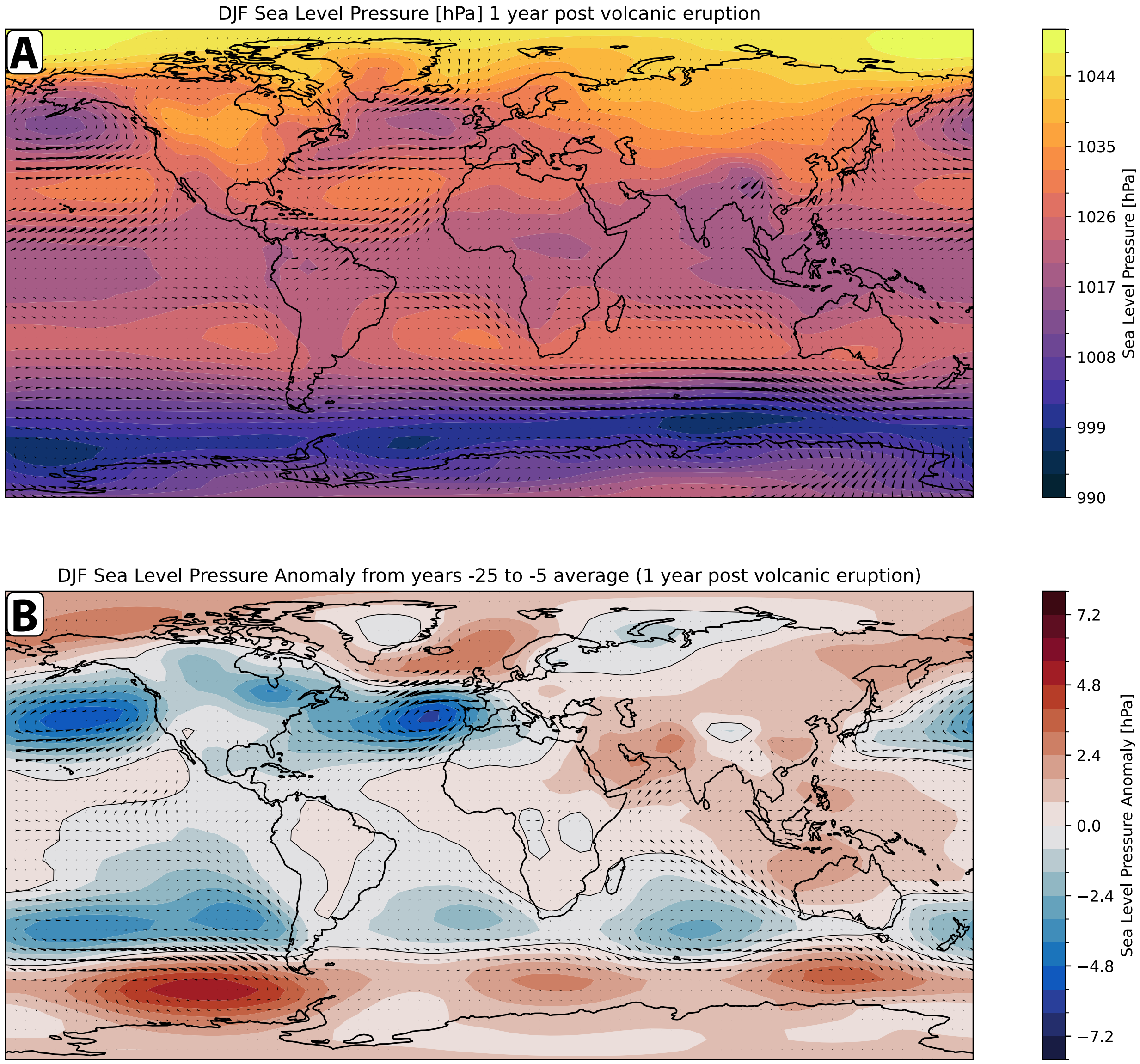

Figure S7: **Post-volcanic global mean sea level pressure response.** (**A**) The Mean Sea Level Pressure climatology and surface wind stress fields one year after the volcanic event. (**B**) The Mean Sea Level Pressure anomaly and surface wind stress anomaly of the glacial climate system one year after an equatorial 230 Tg volcanic aerosol eruption. The anomaly is plotted relative to the mean climatology before the event (years -25 to 5 averaged). This anomalous pattern suggests that planetary wave forcing, originating from changes in the Pacific, induces sea level pressure changes in the North Atlantic, which reorganize surface wind patterns and result in a weakening of the NAO and also affecting North Atlantic Deep Water Formation, through changes in the strength of the North Atlantic sub-polar gyre.

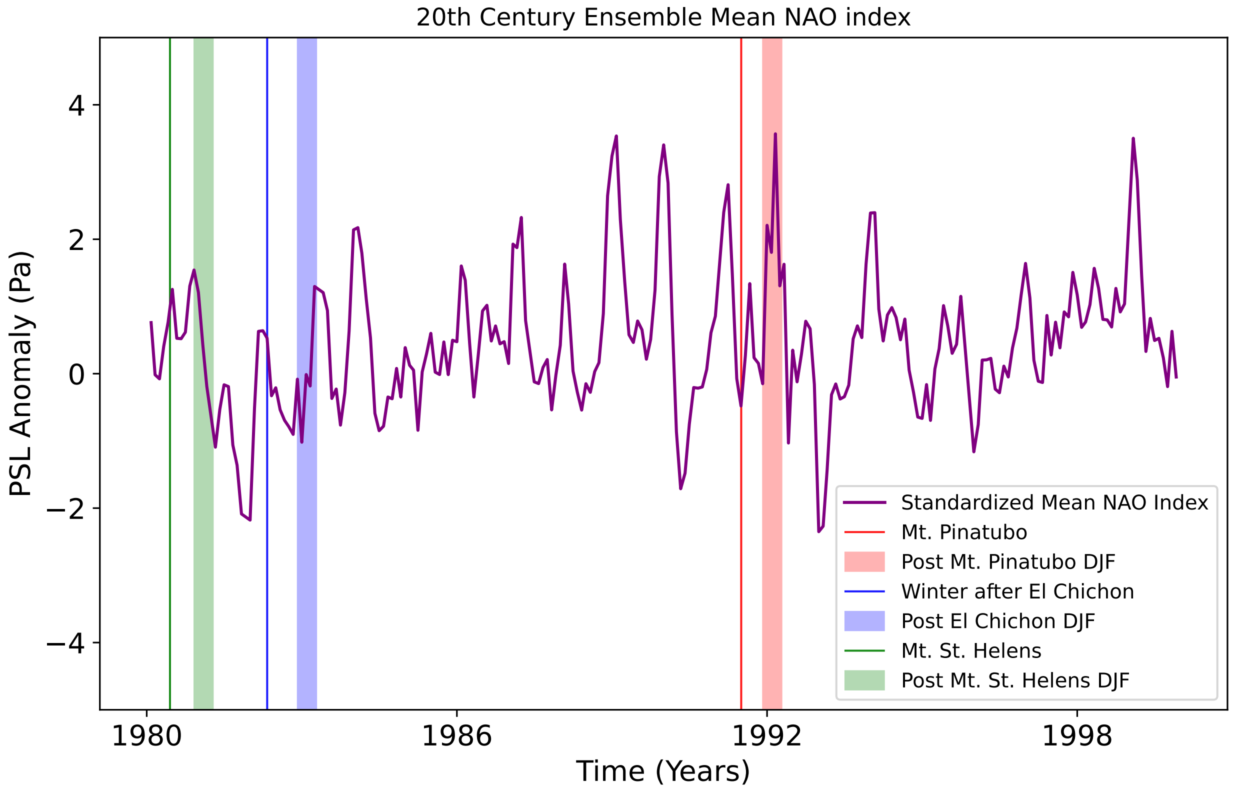

Figure S8: **CCSM4 late 20th century NAO.** Modern response of the NAO in the modern model ensemble to late 20th century volcanic eruptions. The mean modern 20th century ensemble is from Fig. S4. A positive NAO index indicates anomalously low pressure in the Icelandic Low region and anomalously high pressure in the Azores region. Niño 3.4 region (5°N-5°S, 170°W-120°W) NAO index = Azores High Icelandic Low: Icelandic Low region: 60°N-65°N, 20°W-30°W; Azores High region: 35°N-40°N, 20°W-30°W

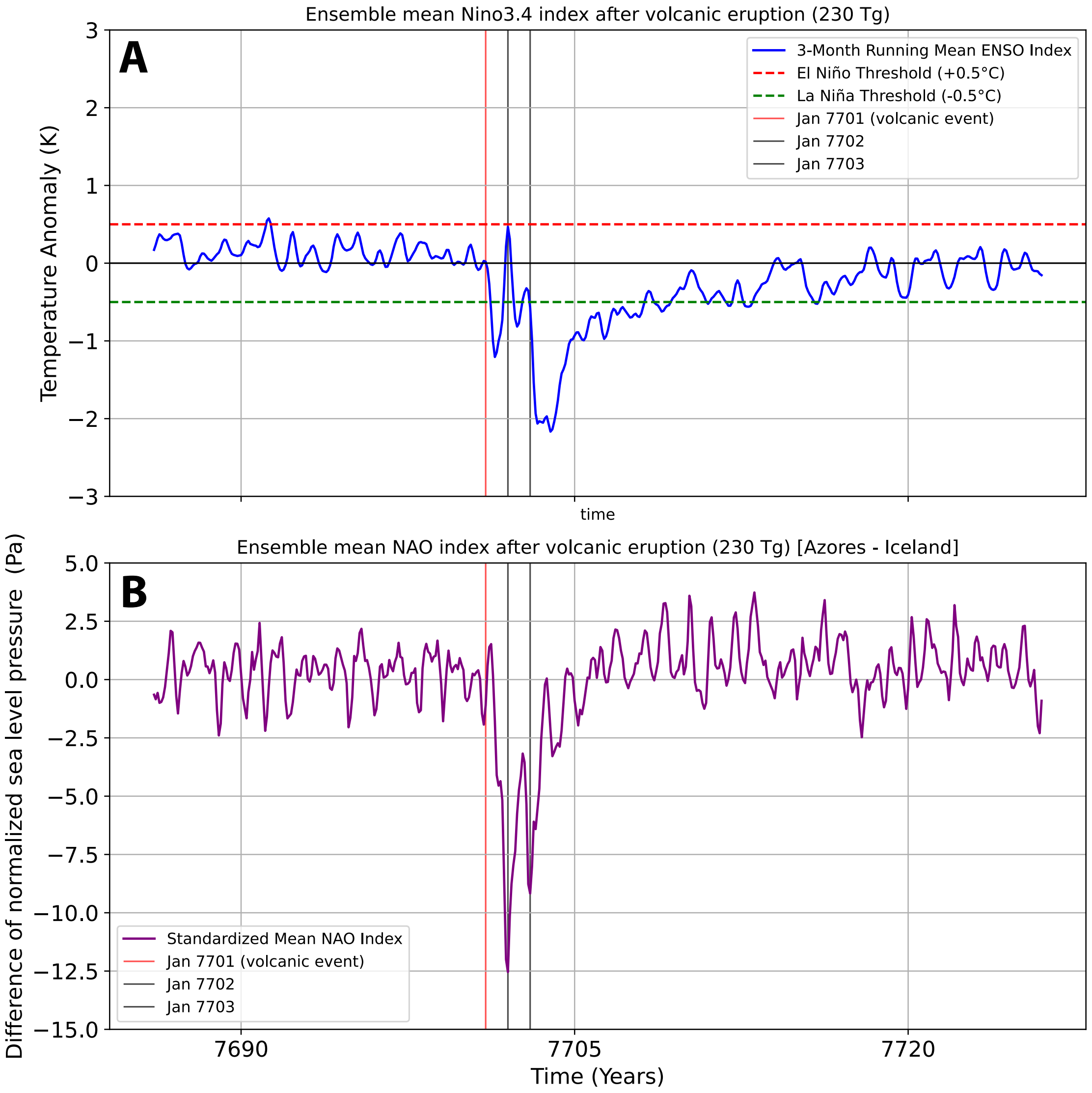

Figure S9: **Glacial CCSM4 post-volcanic ENSO and NAO response.** Standardized (**A**) ENSO and (**B**) NAO indexes after the equatorial 230 Tg volcanic aerosol eruption. In the top panel there is a general cooling after the volcanic event. In the first January after the volcanic event, one year later, there is a strong El Niño anomaly, but the warming signal is superimposed on top of the strong multi-year global cooling induced by the very large volcanic eruption. The second January after the volcanic eruption is also an El Niño year. After the volcanic event, the NAO index indicates anomalously high pressure in the Icelandic Low region and anomalously low pressure in the Azores region. Niño 3.4 region (5°N-5°S, 170°W-120°W); Icelandic Low region: 60°N-65°N, 20°W-30°W; Azores High region: 35°N-40°N, 20°W-30°W

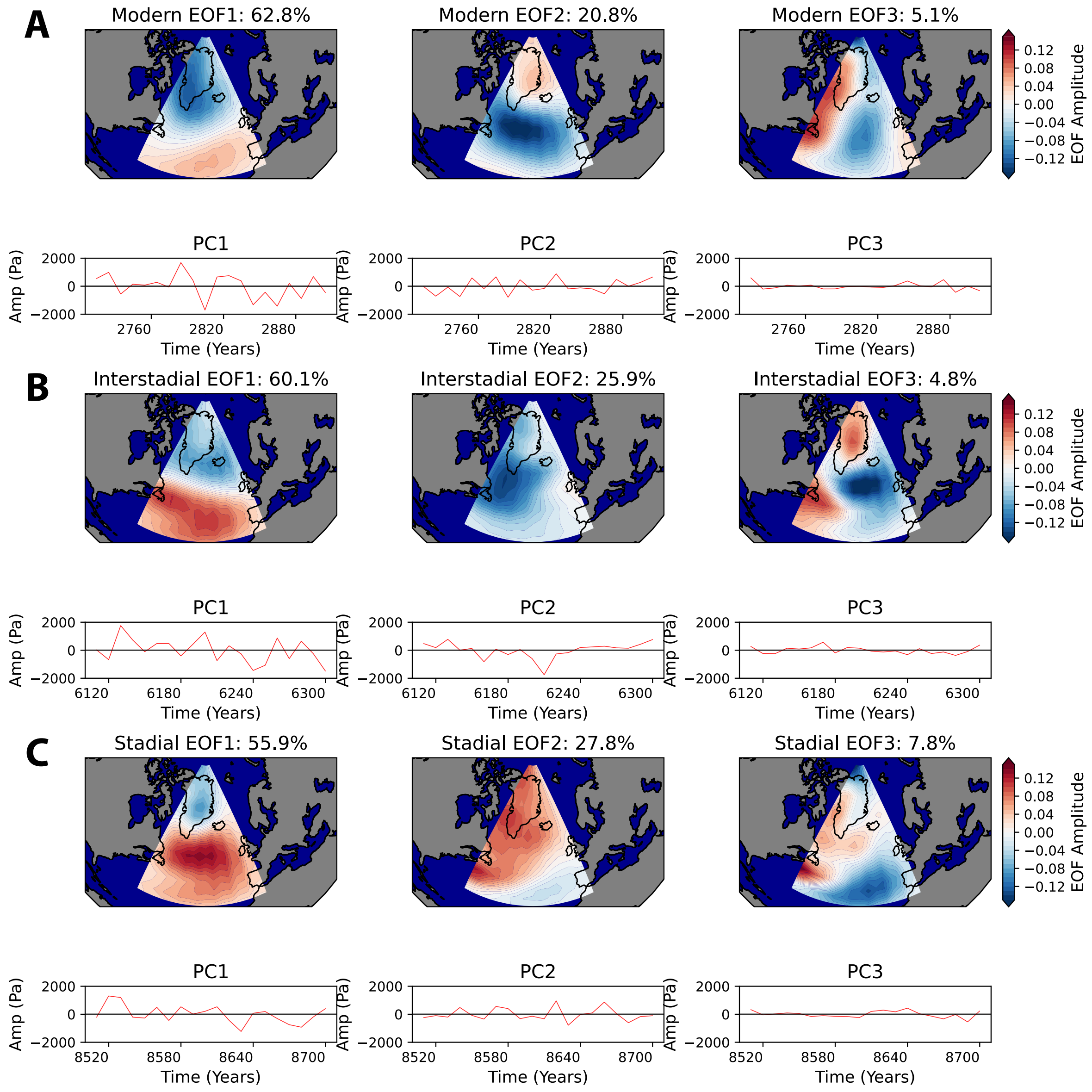

Figure S10: **CCSM4 NAO EOF analysis without volcanism.** The December-January-February (DJF) NAO Empirical Orthogonal Function (EOF) analysis for the (**A**) modern pre-industrial, (**B**) glacial interstadial and (**C**) glacial stadial periods (no volcanism). The first EOF pattern which captures at least half the variance in the simulations has a classic NAO structure. The modern EOF1 mode shows an amplitude of approximately half that seen in the glacial EOF1 mode. EOF2 captures approximately a quarter of the variance in the simulations but diverges between the modern and glacial period. EOF2 in the modern period has a bimodal distribution where the stadial and interstadial are unimodal and are generally of opposite phase.

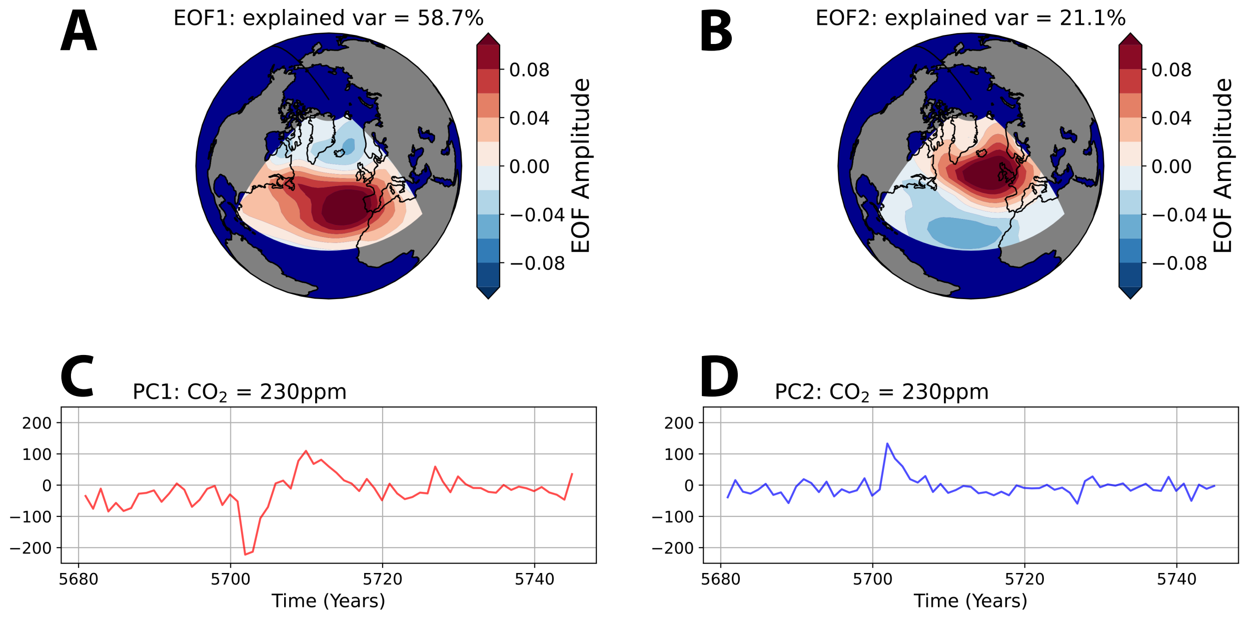

Figure S11: **Glacial CCSM4 NAO EOF analysis with volcanism.** The December-January-February (DJF) NAO due to an equatorial 230 Tg volcanic aerosol eruption in an experiment with an eruption occurring at year 5701 in a glacial D-O simulation. The NAO is captured in the (**A**) first EOF, which explains 58% of the variance. (**B**) The second EOF explains 21% of the variance. (**C, D**) The initial response is a weakening of the NAO index immediately after the volcanic event. Notably, following the weakening response, a re-strengthening of the NAO occurs, which may be linked to the expansion of sea ice in the North Atlantic.

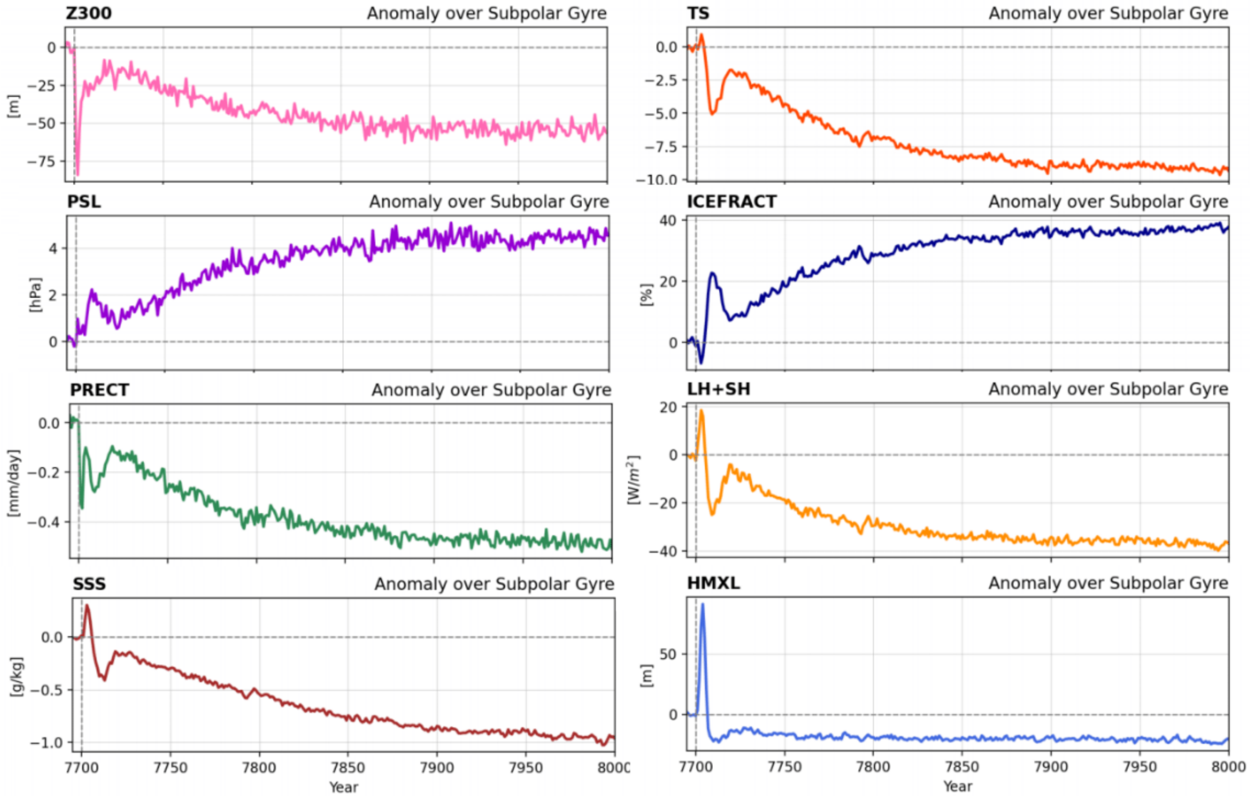

Figure S12: **North Atlantic sub-polar gyre post-volcanic multi-decadal variability.** Ensemble mean climatological anomalies resulting from an equatorial 230 Tg volcanic aerosol forcing at year 7700 are compared with an ensemble of simulations without volcanic forcing over the 300 years following the event (years 7700 to 8000). Plotted anomalies include 300 mb geopotential height (Z300), mean sea level pressure (PSL), total precipitation (PRECT), sea surface salinity (SSS), surface temperature (TS), sea ice concentration (ICEFRACT), combined latent and sensible heat flux (LH+SH), and mixed layer depth (HMXL).

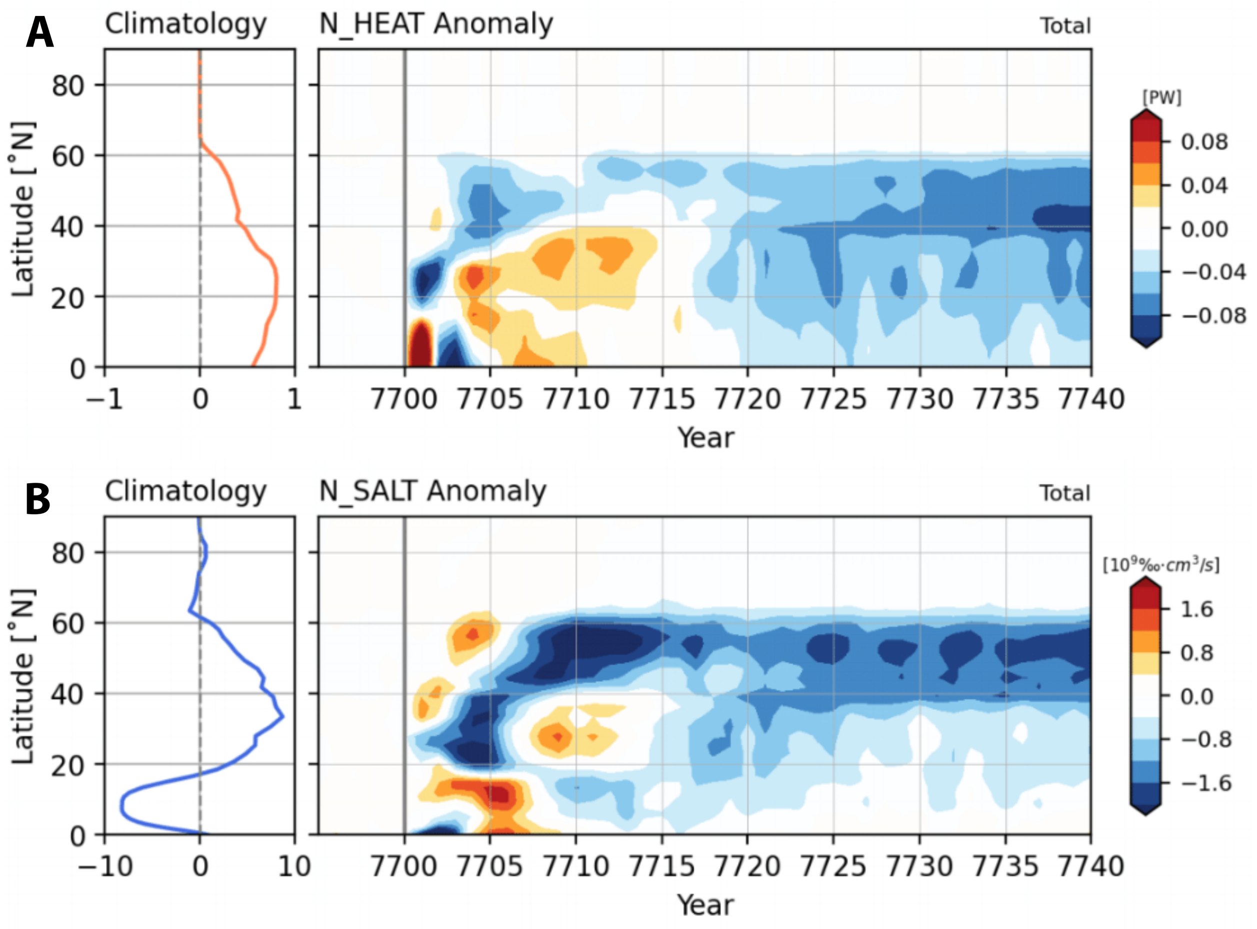

Figure S13: **North Atlantic heat and salt transports.** The mean climatological North Atlantic (**A**) ocean heat and (**B**) salt transport is shown on the left. Anomalies in North Atlantic ocean heat transport (N_HEAT) and salt transport (N_SALT) over the 40 years (right) following an equatorial 230 Tg volcanic aerosol eruption are displayed for the ensemble mean of experiments with volcanic forcing, compared to those without volcanic forcing. The increase in AMOC following the volcanic event is correlated with enhanced northward ocean heat transport.

REFERENCES

1. W. Dansgaard, S. J. Johnsen, J. Moller, C. C. Langway Jr., One thousand centuries of climatic record from Camp Century on the Greenland Ice Sheet. *Science* **166**, 377–380 (1969).

2. S. O. Rasmussen, M. Bigler, S. P. Blockley, T. Blunier, S. L. Buchardt, H. B. Clausen, I. Cvijanovic, D. Dahl-Jensen, S. J. Johnsen, H. Fischer, V. Gkinis, M. Guillevic, W. Z. Hoek, J. J. Lowe, J. B. Pedro, T. Popp, I. K. Seierstad, J. P. Steffensen, A. M. Svensson, P. Vallelonga, B. M. Vinther, M. J. Walker, J. J. Wheatley, M. Winstrup, A stratigraphic framework for abrupt climatic changes during the Last Glacial period based on three synchronized Greenland ice-core records: Refining and extending the INTIMATE event stratigraphy. *Quat. Sci. Rev.* **106**, 14–28 (2014).

3. H. Stommel, Thermohaline convection with two stable regimes of flow. *Tellus* **13**, 224–230 (1961).

4. C. Li, A. Born, Coupled atmosphere-ice-ocean dynamics in Dansgaard-Oeschger events. *Quat. Sci. Rev.* **203**, 1–20 (2019).

5. S. Rahmstorf, Is the Atlantic overturning circulation approaching a tipping point? *Oceanography* **37**, 16–29 (2024).

6. J. P. Steffensen, K. K. Andersen, M. Bigler, H. B. Clausen, D. Dahl-Jensen, H. Fischer, K. Goto-Azuma, M. Hansson, S. J. Johnsen, J. Jouzel, V. Masson-Delmotte, T. Popp, S. O. Rasmussen, R. Rothlisberger, U. Ruth, B. Stauffer, M.-L. Siggaard-Andersen, A. E. Sveinbjornsdottir, A. Svensson, J. W. C. White, High-resolution Greenland Ice Core Data show abrupt climate change happens in few years. *Science* **321**, 680–684 (2008).

7. T. M. Lenton, J. Rockström, O. Gaffney, S. Rahmstorf, K. Richardson, W. Steffen, H. J. Schellnhuber, Climate tipping points — too risky to bet against. *Nature* **575**, 592–595 (2019).

8. L. G. Henry, J. F. McManus, W. B. Curry, N. L. Roberts, A. M. Piotrowski, L. D. Keigwin, North Atlantic ocean circulation and abrupt climate change during the last glaciation. *Science* **353**, 470–474 (2016).

9. R. M. van Westen, H. A. Dijkstra, Persistent climate model biases in the Atlantic Ocean's freshwater transport. *Ocean Sci.* **20**, 549–567 (2024).

10. I. Bethke, S. Outten, O. H. Otterå, E. Hawkins, S. Wagner, M. Sigl, P. Thorne, Potential volcanic impacts on future climate variability. *Nat. Clim. Chang.* **7**, 799–805 (2017).

11. J. Pedro, C. Andersson, G. Vettoretti, A. Voelker, C. Waelbroeck, T. Dokken, M. Jensen, S. Rasmussen, E. Sessford, M. Jochum, K. Nisancioglu, Dansgaard-Oeschger and Heinrich event temperature anomalies in the North Atlantic set by sea ice, frontal position and thermocline structure. *Quat. Sci. Rev.* **289**, 107599 (2022).

12. T. L. Rasmussen, E. Thomsen, The role of the North Atlantic Drift in the millennial timescale glacial climate fluctuations. *Palaeogeogr. Palaeoclimatol. Palaeoecol.* **210**, 101–116 (2004).

13. T. M. Dokken, K. H. Nisancioglu, C. Li, D. S. Battisti, C. Kissel, Dansgaard-Oeschger cycles: Interactions between ocean and sea ice intrinsic to the Nordic seas. *Paleoceanography* **28**, 491–502 (2013).

14. G. Vettoretti, W. R. Peltier, Thermohaline instability and the formation of glacial North Atlantic super polynyas at the onset of Dansgaard-Oeschger warming events. *Geophys. Res. Lett.* **43**, 5336–5344 (2016).

15. Y. Kuniyoshi, A. Abe-Ouchi, S. Sherriff-Tadano, W. Chan, F. Saito, Effect of climatic precession on Dansgaard-Oeschger-like oscillations. *Geophys. Res. Lett.* **49**, e2021GL095695 (2022).

16. G. Vettoretti, W. R. Peltier, Fast physics and slow physics in the nonlinear Dansgaard–Oeschger relaxation oscillation. *J. Clim.* **31**, 3423–3449 (2018).

17. X. Zhang, S. Barker, G. Knorr, G. Lohmann, R. Drysdale, Y. Sun, D. Hodell, F. Chen, Direct astronomical influence on abrupt climate variability. *Nat. Geosci.* **14**, 819–826 (2021).

18. X. Zhang, G. Knorr, G. Lohmann, S. Barker, Abrupt North Atlantic circulation changes in response to gradual CO_2 forcing in a glacial climate state. *Nat. Geosci.* **10**, 518–523 (2017).

19. G. Vettoretti, P. Ditlevsen, M. Jochum, S. O. Rasmussen, Atmospheric CO_2 control of spontaneous millennial-scale ice age climate oscillations. *Nat. Geosci.* **15**, 300–306 (2022).

20. X. Zhang, G. Lohmann, G. Knorr, C. Purcell, Abrupt glacial climate shifts controlled by ice sheet changes. *Nature* **512**, 290–294 (2014).

21. I. Malmierca-Vallet, L. C. Sime, the D–O community members, Dansgaard–Oeschger events in climate models: Review and baseline Marine Isotope Stage 3 (MIS3) protocol. *Clim. Past* **19**, 915–942 (2023).

22. H. Kleppin, M. Jochum, B. Otto-Bliesner, C. A. Shields, S. Yeager, Stochastic atmospheric forcing as a cause of Greenland climate transitions. *J. Clim.* **28**, 7741–7763 (2015).

23. M. Klockmann, U. Mikolajewicz, J. Marotzke, Two AMOC states in response to decreasing greenhouse gas concentrations in the coupled climate model MPI-ESM. *J. Clim.* **31**, 7969–7984 (2018).

24. J. T. Andrews, A. H. Voelker, "Heinrich events" (& sediments): A history of terminology and recommendations for future usage. *Quat. Sci. Rev.* **187**, 31–40 (2018).

25. M. Klockmann, U. Mikolajewicz, H. Kleppin, J. Marotzke, Coupling of the subpolar gyre and the overturning circulation during abrupt glacial climate transitions. *Geophys. Res. Lett.* **47**, e2020GL090361 (2020).

26. H. Zhang, H. Cheng, C. Spötl, X. Zhang, F. W. Cruz, A. Sinha, A. S. Auler, N. M. Stríkis, X. Wang, G. Kathayat, X. Li, H. Li, C. Pérez-Mejías, Y. Cai, Y. Ning, R. L. Edwards, Gradual south-north climate transition in the Atlantic realm within the younger dryas. *Geophys. Res. Lett.* **48**, e2021GL092620 (2021).

27. A. Ganopolski, S. Rahmstorf, Abrupt glacial climate changes due to stochastic resonance. *Phys. Rev. Lett.* **88**, 038501 (2002).

28. F. Kwasniok, Analysis and modelling of glacial climate transitions using simple dynamical systems. *Philos. Trans. A Math. Phys. Eng. Sci.* **371**, 20110472 (2013).

29. T. Mitsui, M. Crucifix, Influence of external forcings on abrupt millennial-scale climate changes: A statistical modelling study. *Clim. Dyn.* **48**, 2729–2749 (2017).

30. A. Roberts, R. Saha, Relaxation oscillations in an idealized ocean circulation model. *Clim. Dyn.* **48**, 2123–2134 (2017).

31. K. Riechers, G. Gottwald, N. Boers, Glacial abrupt climate change as a multiscale phenomenon resulting from monostable excitable dynamics. *J. Clim.* **37**, 2741–2763 (2024).

32. J. O. Melcher, S. Halkjær, P. Ditlevsen, P. L. Langen, G. Vettoretti, S. O. Rasmussen, A novel conceptual model for Dansgaard–Oeschger event dynamics based on ice-core data. *Clim. Past* **21**, 115–132 (2025).

33. L.-P. Nadeau, M. F. Jansen, S. Gregorio, A simple model for global temperature control on Dansgaard Oeschger oscillations. *J. Clim.* **38**, 3169–3184 (2025).

34. J. R. Bray, Volcanism and glaciation during the past 40 millennia. *Nature* **252**, 679–680 (1974).

35. J. U. Baldini, R. J. Brown, J. N. McElwaine, Was millennial scale climate change during the Last Glacial triggered by explosive volcanism? *Sci. Rep.* **5**, 17442 (2015).

36. R. C. Bay, N. Bramall, P. B. Price, Bipolar correlation of volcanism with millennial climate change. *Proc. Natl. Acad. Sci. U.S.A.* **101**, 6341–6345 (2004).

37. P. Abbott, U. Niemeier, C. Timmreck, F. Riede, J. McConnell, M. Severi, H. Fischer, A. Svensson, M. Toohey, F. Reinig, M. Sigl, Volcanic climate forcing preceding the inception of the younger dryas: Implications for tracing the Laacher See eruption. *Quat. Sci. Rev.* **274**, 107260 (2021).

38. A. Svensson, D. Dahl-Jensen, J. P. Steffensen, T. Blunier, S. O. Rasmussen, B. M. Vinther, P. Vallelonga, E. Capron, V. Gkinis, E. Cook, H. A. Kjær, R. Muscheler, S. Kipfstuhl, F. Wilhelms, T. F. Stocker, H. Fischer, F. Adolphi, T. Erhardt, M. Sigl, A. Landais, F. Parrenin, C. Buizert, J. R. McConnell, M. Severi, R. Mulvaney, M. Bigler, Bipolar volcanic synchronization of abrupt

climate change in Greenland and Antarctic ice cores during the last glacial period. *Clim. Past* **16**, 1565–1580 (2020).

39. E. C. Corrick, R. N. Drysdale, J. C. Hellstrom, E. Capron, S. O. Rasmussen, X. Zhang, D. Fleitmann, I. Couchoud, E. Wolff, Synchronous timing of abrupt climate changes during the last glacial period. *Science* **369**, 963–969 (2020).

40. C. S. Lane, A. Brauer, S. P. Blockley, P. Dulski, Volcanic ash reveals time-transgressive abrupt climate change during the younger dryas. *Geology* **41**, 1251–1254 (2013).

41. B. Scaillet, C. Oppenheimer, On the budget and atmospheric fate of sulfur emissions from large volcanic eruptions. *Geophys. Res. Lett.* **51**, e2023GL107180 (2024).

42. T. Erhardt, E. Capron, S. O. Rasmussen, S. Schüpbach, M. Bigler, F. Adolphi, H. Fischer, Decadal-scale progression of the onset of Dansgaard–Oeschger warming events. *Clim. Past* **15**, 811–825 (2019).

43. S. K. Brown, H. S. Crosweller, R. S. J. Sparks, E. Cottrell, N. I. Deligne, N. O. Guerrero, L. Hobbs, K. Kiyosugi, S. C. Loughlin, L. Siebert, S. Takarada, Characterisation of the quaternary eruption record: Analysis of the large magnitude explosive volcanic eruptions (LaMEVE) database. *J. Appl. Volcanol.* **3**, 5 (2014).

44. J. Rougier, S. R. Sparks, K. V. Cashman, Global recording rates for large eruptions. *J. Appl. Volcanol.* **5**, 11 (2016).

45. C. Buizert, A. Schmittner, Southern Ocean control of glacial AMOC stability and Dansgaard-Oeschger interstadial duration. *Paleoceanography* **30**, 1595–1612 (2015).

46. J. Lohmann, A. Svensson, Ice core evidence for major volcanic eruptions at the onset of Dansgaard–Oeschger warming events. *Clim. Past* **18**, 2021–2043 (2022).

47. J. Lohmann, J. Lin, B. M. Vinther, S. O. Rasmussen, A. Svensson, State-dependent impact of major volcanic eruptions observed in ice-core records of the last glacial period. *Clim. Past* **20**, 313–333 (2024).

48. J. Lin, A. Svensson, C. S. Hvidberg, J. Lohmann, S. Kristiansen, D. Dahl-Jensen, J. P. Steffensen, S. O. Rasmussen, E. Cook, H. A. Kjær, B. M. Vinther, H. Fischer, T. Stocker, M. Sigl, M. Bigler, M. Severi, *R. Traversi*, R. Mulvaney, Magnitude, frequency and climate forcing of global volcanism during the last glacial period as seen in Greenland and Antarctic ice cores (60–9 ka). *Clim. Past* **18**, 485–506 (2022).

49. M. Sigl, M. Toohey, J. R. McConnell, J. Cole-Dai, M. Severi, Volcanic stratospheric sulfur injections and aerosol optical depth during the Holocene (past 11500 years) from a bipolar ice-core array. *Earth Syst. Sci. Data* **14**, 3167–3196 (2022).

50. H. M. Innes, W. Hutchison, M. Sigl, L. Crick, P. M. Abbott, M. Bigler, N. J. Chellman, S. M. Davies, S. Kutterolf, J. R. McConnell, M. Severi, R. S. J. Sparks, A. Svensson, E. W. Wolff, J. W. B. Rae, A. Burke, Ice core evidence for the Los Chocoyos supereruption disputes millennial-scale climate impact. *Commun. Earth Environ.* **6**, 137 (2025).

51. J. Lin, P. M. Abbott, M. Sigl, J. P. Steffensen, R. Mulvaney, M. Severi, A. Svensson, Bipolar ice-core records constrain possible dates and global radiative forcing following the ∼74 ka Toba eruption. *Quat. Sci. Rev.* **312**, 108162 (2023).

52. A. Robock, Volcanic eruptions and climate. *Rev. Geophys.* **38**, 191–219 (2000).

53. M. Sigl, M. Winstrup, J. R. McConnell, K. C. Welten, G. Plunkett, F. Ludlow, U. Büntgen, M. Caffee, N. Chellman, D. Dahl-Jensen, H. Fischer, S. Kipfstuhl, C. Kostick, O. J. Maselli, F. Mekhaldi, R. Mulvaney, R. Muscheler, D. R. Pasteris, J. R. Pilcher, M. Salzer, S. Schüpbach, J. P. Steffensen, B. M. Vinther, T. E. Woodruff, Timing and climate forcing of volcanic eruptions for the past 2,500 years. *Nature* **523**, 543–549 (2015).

54. S. McGregor, A. Timmermann, The effect of explosive tropical volcanism on ENSO. *J. Clim.* **24**, 2178–2191 (2011).

55. F. S. R. Pausata, L. Chafik, R. Caballero, D. S. Battisti, Impacts of high-latitude volcanic eruptions on ENSO and AMOC. *Proc. Natl. Acad. Sci. U.S.A.* **112**, 13784–13788 (2015).

56. F. S. R. Pausata, Y. Zhao, D. Zanchettin, R. Caballero, D. S. Battisti, Revisiting the mechanisms of ENSO response to tropical volcanic eruptions. *Geophys. Res. Lett.* **50**, e2022GL102183 (2023).

57. M. Khodri, T. Izumo, J. Vialard, S. Janicot, C. Cassou, M. Lengaigne, J. Mignot, G. Gastineau, E. Guilyardi, N. Lebas, A. Robock, M. J. McPhaden, Tropical explosive volcanic eruptions can trigger El Niño by cooling tropical Africa. *Nat. Commun.* **8**, 778 (2017).

58. M. M. Dogar, L. Hermanson, A. A. Scaife, D. Visioni, M. Zhao, I. Hoteit, H.-F. Graf, M. A. Dogar, M. Almazroui, M. Fujiwara, A review of El Niño Southern Oscillation linkage to strong volcanic eruptions and post-volcanic winter warming. *Earth Syst. Environ.* **7**, 15–42 (2023).

59. M. M. Dogar, M. Fujiwara, M. Zhao, M. Ohba, Y. Kosaka, ENSO and NAO linkage to strong volcanism and associated post-volcanic high-latitude winter warming. *Geophys. Res. Lett.* **51**, e2023GL106114 (2024).

60. S. Brönnimann, J. Franke, S. U. Nussbaumer, H. J. Zumbühl, D. Steiner, M. Trachsel, G. C. Hegerl, A. Schurer, M. Worni, A. Malik, J. Flückiger, C. C. Raible, Last phase of the Little Ice Age forced by volcanic eruptions. *Nat. Geosci.* **12**, 650–656 (2019).

61. M. Gupta, J. Marshall, The climate response to multiple volcanic eruptions mediated by ocean heat uptake: Damping processes and accumulation potential. *J. Clim.* **31**, 8669–8687 (2018).

62. F. Lehner, A. Born, C. C. Raible, T. F. Stocker, Amplified inception of European Little Ice Age by sea ice–ocean–atmosphere feedbacks. *J. Clim.* **26**, 7586–7602 (2013).

63. G. H. Miller, Á. Geirsdóttir, Y. Zhong, D. J. Larsen, B. L. Otto-Bliesner, M. M. Holland, D. A. Bailey, K. A. Refsnider, S. J. Lehman, J. R. Southon, C. Anderson, H. Björnsson, T. Thordarson, Abrupt onset of the Little Ice Age triggered by volcanism and sustained by sea-ice/ocean feedbacks. *Geophys. Res. Lett.* **39**, L02708 (2012).

64. J. Slawinska, A. Robock, Impact of volcanic eruptions on decadal to centennial fluctuations of Arctic Sea Ice extent during the last millennium and on initiation of the Little Ice Age. *J. Clim.* **31**, 2145–2167 (2018).

65. J. A. Church, N. J. White, J. M. Arblaster, Significant decadal-scale impact of volcanic eruptions on sea level and ocean heat content. *Nature* **438**, 74–77 (2005).

66. D. Swingedouw, P. Ortega, J. Mignot, E. Guilyardi, V. Masson-Delmotte, P. G. Butler, M. Khodri, R. Séférian, Bidecadal North Atlantic ocean circulation variability controlled by timing of volcanic eruptions. *Nat. Commun.* **6**, 6545 (2015).

67. Y. Zhong, G. H. Miller, B. L. Otto-Bliesner, M. M. Holland, D. A. Bailey, D. P. Schneider, A. Geirsdottir, Centennial-scale climate change from decadally-paced explosive volcanism: A coupled sea ice-ocean mechanism. *Clim. Dyn.* **37**, 2373–2387 (2011).

68. E. van Dijk, J. Jungclaus, S. Lorenz, C. Timmreck, K. Krüger, Was there a volcanic-induced long-lasting cooling over the Northern Hemisphere in the mid-6th–7th century? *Clim. Past* **18**, 1601–1623 (2022).

69. D. Zanchettin, C. Timmreck, H.-F. Graf, A. Rubino, S. Lorenz, K. Lohmann, K. Krüger, J. H. Jungclaus, Bi-decadal variability excited in the coupled ocean–atmosphere system by strong tropical volcanic eruptions. *Clim. Dyn.* **39**, 419–444 (2012).

70. L. Chafik, N. P. Holliday, S. Bacon, T. Rossby, Irminger Sea is the center of action for subpolar AMOC variability. *Geophys. Res. Lett.* **49**, e2022GL099133 (2022).

71. B. Orihuela-Pinto, A. Santoso, M. H. England, A. S. Taschetto, Coupled feedbacks from the tropical Pacific to the Atlantic Meridional Overturning Circulation. *Geophys. Res. Lett.* **50**, e2023GL103250 (2023).

72. G. Stenchikov, T. L. Delworth, V. Ramaswamy, R. J. Stouffer, A. Wittenberg, F. Zeng, Volcanic signals in oceans. *J. Geophys. Res. Atmos.* **114**, D16104 (2009).

73. M. Erez, O. Adam, Energetic constraints on the time-dependent response of the ITCZ to volcanic eruptions. *J. Clim.* **34**, 9989–10006 (2021).

74. E. J. C. van Dijk, J. Jungclaus, M. Sigl, C. Timmreck, K. Krüger, High-frequency climate forcing causes prolonged cold periods in the Holocene. *Commun. Earth Environ.* **5**, 242 (2024).

75. O. H. Otterå, M. Bentsen, H. Drange, L. Suo, External forcing as a metronome for Atlantic multidecadal variability. *Nat. Geosci.* **3**, 688–694 (2010).

76. G. L. Stenchikov, I. Kirchner, A. Robock, H.-F. Graf, J. C. Antuña, R. G. Grainger, A. Lambert, L. Thomason, Radiative forcing from the 1991 Mount Pinatubo volcanic eruption. *J. Geophys. Res. Atmos.* **103**, 13837–13857 (1998).

77. M. Stoffel, M. Khodri, *C. Corona*, S. Guillet, V. Poulain, S. Bekki, J. Guiot, B. H. Luckman, C. Oppenheimer, N. Lebas, M. Beniston, V. Masson-Delmotte, Estimates of volcanic-induced cooling in the Northern Hemisphere over the past 1,500 years. *Nat. Geosci.* **8**, 784–788 (2015).

78. C. M. Vidal, N. Métrich, J.-C. Komorowski, I. Pratomo, A. Michel, N. Kartadinata, V. Robert, F. Lavigne, The 1257 Samalas eruption (Lombok, Indonesia): The single greatest stratospheric gas release of the Common Era. *Sci. Rep.* **6**, 34868 (2016).

79. C. Gao, A. Robock, C. Ammann, Volcanic forcing of climate over the past 1500 years: An improved ice core-based index for climate models. *J. Geophys. Res. Atmos.* **113**, D23111 (2008).

80. C. A. Shields, D. A. Bailey, G. Danabasoglu, M. Jochum, J. T. Kiehl, S. Levis, S. Park, The low-resolution CCSM4. *J. Clim.* **25**, 3993–4014 (2012).

81. B. M. Harris, E. J. Highwood, A simple relationship between volcanic sulfate aerosol optical depth and surface temperature change simulated in an atmosphere-ocean general circulation model. *J. Geophys. Res. Atmos.* **116**, D05109 (2011).

82. C. Timmreck, Modeling the climatic effects of large explosive volcanic eruptions. *WIREs Clim. Chang.* **3**, 545–564 (2012).

83. F. S. R. Pausata, A. Grini, R. Caballero, A. Hannachi, Ø. Seland, High-latitude volcanic eruptions in the Norwegian Earth System Model: The effect of different initial conditions and of the ensemble size. *Tellus B* **67**, 26728 (2015).

84. S. M. Davies, P. G. Albert, A. J. Bourne, S. Owen, A. Svensson, M. S. M. Bolton, E. Cook, B. J. L. Jensen, G. Jones, V. V. Ponomareva, T. Suzuki, Exploiting the Greenland volcanic ash

repository to date caldera-forming eruptions and widespread isochrons during the Holocene. *Quat. Sci. Rev.* **334**, 108707 (2024).

85. N. W. Dunbar, N. A. Iverson, A. R. Van Eaton, M. Sigl, B. V. Alloway, A. V. Kurbatov, L. G. Mastin, J. R. McConnell, C. J. N. Wilson, New Zealand supereruption provides time marker for the Last Glacial Maximum in Antarctica. *Sci. Rep.* **7**, 12238 (2017).

86. C. J. N. Wilson, G. F. Cooper, K. J. Chamberlain, S. J. Barker, M. L. Myers, F. Illsley-Kemp, J. Farrell, No single model for supersized eruptions and their magma bodies. *Nat. Rev. Earth Environ.* **2**, 610–627 (2021).

87. M. Toohey, K. Krüger, U. Niemeier, C. Timmreck, The influence of eruption season on the global aerosol evolution and radiative impact of tropical volcanic eruptions. *Atmos. Chem. Phys.* **11**, 12351–12367 (2011).

88. M. Toohey, K. Krüger, C. Timmreck, Volcanic sulfate deposition to Greenland and Antarctica: A modeling sensitivity study. *J. Geophys. Res. Atmos.* **118**, 4788–4800 (2013).

89. Z. Zhuo, H. F. Fuglestvedt, M. Toohey, K. Krüger, Initial atmospheric conditions control transport of volcanic volatiles, forcing and impacts. *Atmos. Chem. Phys.* **24**, 6233–6249 (2024).

90. T. F. Stocker, S. J. Johnsen, A minimum thermodynamic model for the bipolar seesaw. *Paleoceanography* **18**, 1087 (2003).

91. P. Cessi, A simple box model of stochastically forced thermohaline flow. *J. Phys. Oceanogr.* **24**, 1911–1920 (1994).

92. A. M. Iwi, L. Hermanson, K. Haines, R. T. Sutton, Mechanisms linking volcanic aerosols to the Atlantic Meridional Overturning Circulation. *J. Clim.* **25**, 3039–3051 (2012).

93. V. Dakos, M. Scheffer, E. H. van Nes, V. Brovkin, V. Petoukhov, H. Held, Slowing down as an early warning signal for abrupt climate change. *Proc. Natl. Acad. Sci. U.S.A.* **105**, 14308–14312 (2008).

94. T. M. Bury, R. I. Sujith, I. Pavithran, M. Scheffer, T. M. Lenton, M. Anand, C. T. Bauch, Deep learning for early warning signals of tipping points. *Proc. Natl. Acad. Sci. U.S.A.* **118**, e2106140118 (2021).

95. A. Svensson, M. Bigler, T. Blunier, H. B. Clausen, D. Dahl-Jensen, H. Fischer, S. Fujita, K. Goto-Azuma, S. J. Johnsen, K. Kawamura, S. Kipfstuhl, M. Kohno, F. Parrenin, T. Popp, S. O. Rasmussen, J. Schwander, I. Seierstad, M. Severi, J. P. Steffensen, R. Udisti, R. Uemura, P. Vallelonga, B. M. Vinther, A. Wegner, F. Wilhelms, M. Winstrup, Direct linking of Greenland and Antarctic ice cores at the Toba eruption (74 ka BP). *Clim. Past* **9**, 749–766 (2013).

96. A. R. Paine, F. B. Wadsworth, J. U. L. Baldini, Supereruption doublet at a climate transition. *Commun. Earth Environ.* **2**, 219 (2021).

97. E. J. Rohling, J. Yu, D. Heslop, G. L. Foster, B. Opdyke, A. P. Roberts, Sea level and deep-sea temperature reconstructions suggest quasi-stable states and critical transitions over the past 40 million years. *Sci. Adv.* **7**, eabf5326 (2021).

98. B. Bereiter, D. Luthi, M. Siegrist, S. Schupbach, T. F. Stocker, H. Fischer, Mode change of millennial CO_2 variability during the last glacial cycle associated with a bipolar marine carbon seesaw. *Proc. Natl. Acad. Sci. U.S.A.* **109**, 9755–9760 (2012).

99. P. Kindler, M. Guillevic, M. Baumgartner, J. Schwander, A. Landais, M. Leuenberger, Temperature reconstruction from 10 to 120 kyr b2k from the NGRIP ice core. *Clim. Past* **10**, 887–902 (2014).

100. D. Dutta, P. O. Hopcroft, L. S. Andreasen, T. J. Aubry, C. Timmreck, D. Zanchettin, X. Zhang, F. Muschitiello, State-dependent North Atlantic response to volcanic eruption clusters. *Geophys. Res. Lett.* **52**, e2025GL117582 (2025).

101. A. G. Pendergrass, R. Knutti, F. Lehner, C. Deser, B. M. Sanderson, Precipitation variability increases in a warmer climate. *Sci. Rep.* **7**, 17966 (2017).

102. K. Nordling, N. L. S. Fahrenbach, B. H. Samset, Climate variability can outweigh the influence of climate mean changes for extreme precipitation under global warming. *Atmos. Chem. Phys.* **25**, 1659–1684 (2025).

103. P. Ditlevsen, S. Ditlevsen, Warning of a forthcoming collapse of the Atlantic meridional overturning circulation. *Nat. Commun.* **14**, 4254 (2023).

104. R. Chapman, S. Sinet, P. D. L. Ritchie, Tipping mechanisms in a conceptual model of the Atlantic Meridional Overturning Circulation. *Weather* **79**, 316–323 (2024).

105. J. A. Baker, M. J. Bell, L. C. Jackson, R. Renshaw, G. K. Vallis, A. J. Watson, R. A. Wood, Overturning pathways control AMOC weakening in CMIP6 models. *Geophys. Res. Lett.* **50**, e2023GL103381 (2023).

106. W. R. Peltier, G. Vettoretti, Dansgaard-Oeschger oscillations predicted in a comprehensive model of glacial climate: A “kicked” salt oscillator in the Atlantic: Dansgaard-Oeschger Oscillations. *Geophys. Res. Lett.* **41**, 7306–7313 (2014).

107. G. Danabasoglu, S. G. Yeager, Y.-O. Kwon, J. J. Tribbia, A. S. Phillips, J. W. Hurrell, Variability of the Atlantic Meridional Overturning Circulation in CCSM4. *J. Clim.* **25**, 5153–5172 (2012).

108. S. R. Jayne, The impact of abyssal mixing parameterizations in an ocean general circulation model. *J. Phys. Oceanogr.* **39**, 1756–1775 (2009).

109. K. Bryan, L. J. Lewis, A water mass model of the world ocean. *J. Geophys. Res.* **84**, 2503–2517 (1979).

110. S. Wilmes, A. Schmittner, J. A. M. Green, Glacial Ice Sheet extent effects on modeled tidal mixing and the Global Overturning Circulation. *Paleoceanogr. Paleoclimatol.* **34**, 1437–1454 (2019).

111. J. B. Pedro, M. Jochum, C. Buizert, F. He, S. Barker, S. O. Rasmussen, Beyond the bipolar seesaw: Toward a process understanding of interhemispheric coupling. *Quat. Sci. Rev.* **192**, 27–46 (2018).

112. S. Kotz, S. Nadarajah, *Extreme Value Distributions: Theory and Applications* (World Scientific) (2000).

113. S. Coles, *An Introduction to Statistical Modeling of Extreme Values* (Springer Publishing) (2002).

114. G. Vettoretti, W. R. Peltier, Interhemispheric air temperature phase relationships in the nonlinear Dansgaard-Oeschger oscillation. *Geophys. Res. Lett.* **42**, 1180–1189 (2015).

115. K. M. Cuffey, G. D. Clow, E. J. Steig, C. Buizert, T. J. Fudge, M. Koutnik, E. D. Waddington, R. B. Alley, J. P. Severinghaus, Deglacial temperature history of West Antarctica. *Proc. Natl. Acad. Sci. U.S.A.* **113**, 14249–14254 (2016).

116. E. J. Gowan, X. Zhang, S. Khosravi, A. Rovere, P. Stocchi, A. L. C. Hughes, R. Gyllencreutz, J. Mangerud, J.-I. Svendsen, G. Lohmann, A new global ice sheet reconstruction for the past 80 000 years. *Nat. Commun.* **12**, 1199 (2021).

117. Y. Chen, P. Song, X. Chen, G. Lohmann, Glacial AMOC shoaling despite vigorous tidal dissipation: Vertical stratification matters. *Clim. Past* **20**, 2001–2015 (2024).

118. C. M. Ammann, G. A. Meehl, W. M. Washington, C. S. Zender, A monthly and latitudinally varying volcanic forcing dataset in simulations of 20th century climate. *Geophys. Res. Lett.* **30**, 1657 (2003).

119. M. Toohey, M. Sigl, Volcanic stratospheric sulfur injections and aerosol optical depth from 500 BCE to 1900 CE. *Earth Syst. Sci. Data* **9**, 809–831 (2017).

120. R. R. Neely III, A. J. Conley, F. Vitt, J.-F. Lamarque, A consistent prescription of stratospheric aerosol for both radiation and chemistry in the Community Earth System Model (CESM1). *Geosci. Model Dev.* **9**, 2459–2470 (2016).

121. C. Gao, L. Oman, A. Robock, G. L. Stenchikov, Atmospheric volcanic loading derived from bipolar ice cores: Accounting for the spatial distribution of volcanic deposition. *J. Geophys. Res. Atmos.* **112**, D09109 (2007).

122. T. J. Crowley, M. B. Unterman, Technical details concerning development of a 1200 yr proxy index for global volcanism. *Earth Syst. Sci. Data* **5**, 187–197 (2013).

123. J. Hansen, R. Ruedy, M. Sato, K. Lo, Global surface temperature change. *Rev. Geophys.* **48**, RG4004 (2010).

124. N. Lenssen, G. A. Schmidt, M. Hendrickson, P. Jacobs, M. J. Menne, R. Ruedy, A NASA GISTEMPv4 Observational Uncertainty Ensemble. *J. Geophys. Res. Atmos.* **129**, e2023JD040179 (2024).

125. E. Kalnay, M. Kanamitsu, R. Kistler, W. Collins, D. Deaven, L. Gandin, M. Iredell, S. Saha, G. White, J. Woollen, Y. Zhu, M. Chelliah, W. Ebisuzaki, W. Higgins, J. Janowiak, K. C. Mo, C. Ropelewski, J. Wang, A. Leetmaa, R. Reynolds, R. Jenne, D. Joseph, The NCEP/NCAR 40-Year reanalysis project. *Bull. Amer. Meteorol. Soc.* **77**, 437–472 (1996).

126. M. Sato, J. E. Hansen, M. P. McCormick, J. B. Pollack, Stratospheric aerosol optical depths, 1850–1990. *J. Geophys. Res. Atmos.* **98**, 22987–22994 (1993).

127. C. Timmreck, H.-F. Graf, S. J. Lorenz, U. Niemeier, D. Zanchettin, D. Matei, J. H. Jungclaus, T. J. Crowley, Aerosol size confines climate response to volcanic super-eruptions. *Geophys. Res. Lett.* **37**, L24705 (2010).

128. R. Wilson, K. Anchukaitis, K. R. Briffa, U. Büntgen, E. Cook, R. D’Arrigo, N. Davi, J. Esper, D. Frank, B. Gunnarson, G. Hegerl, S. Helama, S. Klesse, P. J. Krusic, H. W. Linderholm, V. Myglan, T. J. Osborn, M. Rydval, L. Schneider, A. Schurer, G. Wiles, P. Zhang, E. Zorita, Last millennium northern hemisphere summer temperatures from tree rings: Part I: The long term context. *Quat. Sci. Rev.* **134**, 1–18 (2016).

129. D. Zanchettin, O. Bothe, H. F. Graf, S. J. Lorenz, J. Luterbacher, C. Timmreck, J. H. Jungclaus, Background conditions influence the decadal climate response to strong volcanic eruptions. *J. Geophys. Res. Atmos.* **118**, 4090–4106 (2013).

130. B. Ellerhoff, M. J. Kirschner, E. Ziegler, M. D. Holloway, L. Sime, K. Rehfeld, Contrasting state-dependent effects of natural forcing on global and local climate variability. *Geophys. Res. Lett.* **49**, e2022GL098335 (2022).

131. L. Marshall, A. Schmidt, M. Toohey, K. S. Carslaw, G. W. Mann, M. Sigl, M. Khodri, C. Timmreck, D. Zanchettin, W. T. Ball, S. Bekki, J. S. A. Brooke, S. Dhomse, C. Johnson, J.-F. Lamarque, A. N. LeGrande, M. J. Mills, U. Niemeier, J. O. Pope, V. Poulain, A. Robock, E. Rozanov, A. Stenke, T. Sukhodolov, S. Tilmes, K. Tsigaridis, F. Tummon, Multi-model comparison of the volcanic sulfate deposition from the 1815 eruption of Mt. Tambora. *Atmos. Chem. Phys.* **18**, 2307–2328 (2018).

132. G. Rivière, A. Laîné, G. Lapeyre, D. Salas-Mélia, M. Kageyama, Links between Rossby Wave Breaking and the North Atlantic Oscillation–Arctic Oscillation in Present-Day and Last Glacial Maximum Climate Simulations. *J. Clim.* **23**, 2987–3008 (2010).
